# Assessing functional benefits of specific apple rootstock/AMF associations within context-dependent frameworks

**DOI:** 10.3389/fpls.2026.1914090

**Published:** 2026-09-24

**Authors:** Chris Cook, Schaefer Buchanan, Wade Heller, Lee Kalcsits, Tracey Somera

**Affiliations:** 1 Department of Horticulture, Tree Fruit Research and Extension Center, Washington State University, Wenatchee, WA, United States; 2 Eastern Regional Research Center, Sustainable Biofuels and Co-Products Research Unit, United States Department of Agriculture (USDA), Agricultural Research Service, Wyndmoor, PA, United States; 3 Physiology and Pathology of Tree Fruits Research Unit, USDA Agricultural Research Service, Wenatchee, WA, United States

**Keywords:** apple replant disease, apple rootstock interactions, arbuscular mycorrhizal fungi, drought stress tolerance, nitrogen assimilation

## Abstract

Arbuscular mycorrhizal fungi (AMF) are root-colonizing endophytes present in most terrestrial ecosystems, including agricultural soils. AMF have been shown to provide a spectrum of benefits to their plant hosts. Their functional benefits can be both environmental-context dependent and fungus-plant-species dependent. How contextual controls govern AMF-mediated benefits is not well-understood, yet this knowledge is key to successful “real-world” application. The objective of this study was to assess contextual benefits of specific apple rootstock/AMF associations, including improved tolerance to water stress, nutrient access, and disease resistance. In conjunction, we sought to better understand how certain context-dependent factors, such as host genotype and soil microbial context influenced anticipated functional benefits. Specifically, in the first experiment, we tested the ability of *Rhizophagus irregularis* to improve water and inorganic nitrogen (^15^NH_4_NO_3_) uptake in G.11 rootstock with (and without) water stress in “live” (replant disease-conducive) vs. pasteurized (pathogen-free) orchard soil. While AMF inoculation did positively influence plant water and nitrogen uptake, these benefits were modulated by watering regime and dependent on soil microbial context. Under replant conditions, pre-colonization of G.11 rootstocks by *R.irregularis* led to significant increases in stomatal conductance in both well-watered and water-limited conditions. By notable comparison, soil pasteurization appeared to negate this benefit. At the same time (in live soil), AMF inoculation was associated with significantly greater total ^15^N isotope tracer recovery under well-watered (but not water-stressed) conditions. The second experiment focused on the role host genotype/AMF species identity plays in reducing pathogen infection in apple roots. The ability of the AMF *Claroideoglomus etunicatum* and *Claroideoglomus claroideum* to reduce infection by the fungal replant pathogen *Rhizoctonia solani* AG-5 in G.41 and G.890 was tested. Findings showed AMF directly limited pathogen establishment. The degree of protective effect varied, however, according to the particular host genotype/AMF species combination. Both experiments establish clear evidence of AMF species directly functioning in beneficial roles with commercially available apple rootstock genotypes and provide insight into specific AMF-rootstock relationships with the potential to promote more resilient and sustainable apple production. The context-dependency of these outcomes highlights the need to move beyond generalized strategies for enhancing mycorrhizal benefits in orchard systems.

## 1 Introduction

It is well known that arbuscular mycorrhizal fungi (AMF; phylum *Glomeromycota*) are widespread soilborne endosymbionts colonizing over 80% of terrestrial plant species (Schüßler et al., 2001; Wang and Qiu, 2006). They have been shown to enhance plant productivity by transferring a variety of mineral nutrients, including phosphorus and nitrogen to their host plant in exchange for photosynthetically derived carbon (Smith, 1982; Cui and Caldwell, 1996; Karandashov and Bucher, 2005). AMF have also been shown to improve plant tissue concentrations of sulfur (Sieh et al., 2013), potassium (Garcia and Zimmermann, 2014), zinc (Lehmann et al., 2014; Ho-Plágaro et al., 2024), copper, and iron (Lehmann and Rillig, 2015). Besides nutritional benefits, AMF can provide plants with protection against a broad range of pathogens and pests, both above and below ground. Roots colonized by AMF are less likely to be colonized by pathogens (Resendes et al., 2008) due to a combination of several mechanisms, including competitive exclusion and AMF-mediated modulation of plant defense responses (Liu et al., 2007; Weng et al., 2022). There is also a growing body of literature that is focused on enhancing crop resilience in response to climatic variability. This literature underscores the role of AMF in abiotic stress resilience, particularly in regard to water stress. While earlier studies questioned the direct involvement of AMF in plant water uptake, and primarily attributed drought tolerance to improved soil structure and nutrient acquisition (Smith et al., 2010), recent microscopic evidence demonstrates AMF hyphae do supply water to host plants (Kakouridis et al., 2022). In addition to direct water absorption, AMF symbiosis can help alleviate drought stress through other protective mechanisms including: aquaporin regulation, antioxidant enhancement, increased phytohormone production, and osmotic regulation (Bahadur et al., 2019).

Although many species of AMF are generalists, and can colonize a range of host plants, a growing body of evidence suggests that plant-AMF preferences exist in many different environments and cropping systems (Sanders, 2003). Furthermore, functional benefits may be fungus and plant-species/cultivar dependent (Klironomos, 2000). Much of the literature describing plant-fungal preferences has been based on host plant identity at the family, genus or species level. However, in modern agricultural systems, including tree fruit orchards, management decisions are frequently made at the cultivar/rootstock level (e.g., rootstock selection). Thus, in terms of optimizing the use of mycorrhizal inoculum in agricultural systems, there is a need for increased understanding of intraspecific plant-AMF interactions. Research in this area includes studies with modern vs. legacy wheat (Hetrick et al., 1993) and tomato cultivars (Steinkellner et al., 2012). More recent studies in apple (Van Horn et al., 2021) grapevine (Noceto et al., 2024) and almond (Vargas Ribera et al., 2025) have demonstrated the effect of rootstock genotype on the composition of the AMF root-associated community. In a different study, which utilized a variety of Geneva apple rootstocks, some AMF species (*Rhizophagus irregularis* and *Claroideoglomus etunicatum*) were better colonizers than others, regardless of rootstock genotype (Cook et al., 2024). Although plant-AMF interactions are complex and are likely influenced by factors including soil properties and environmental conditions, some fungal-host combinations may be more suitable than others for use in nursery/orchard systems.

There is a substantial amount of evidence demonstrating that arbuscular mycorrhizal fungi can benefit plant health; however, the extent to which AMF can be used to enhance agricultural practices in many production systems, including tree fruit, remains unclear (Koziol et al., 2025). This is largely because mycorrhizal-mediated benefits involve complex interactions between several physical and biological factors, including other soil/rhizosphere microorganisms. While many studies utilizing AMF inoculants are conducted in sterilized soils (to isolate the effects of the introduced AMF species without competition from native microorganisms), plant-microbe interactions are significantly more complex in live soil. For instance, mycorrhizal fungi form co-occurrence networks with bacteria and archaea in bulk soil (Yuan et al., 2021) and AMF hyphae can be actively colonized by bacteria (Scheublin et al., 2010; Toljander et al., 2007). In the rhizosphere, mycorrhization helper bacteria (MHBs) can facilitate plant-AMF colonization (Frey-Klett et al., 2007). Considering this, it should be noted that several AMF-mediated functional benefits have been documented in apple.

Improved P uptake (relative to uninoculated controls) was observed in shoots of micropropagated plants of the apple rootstock MM.106 when colonized by *F. mosseae* (Schubert and Lubraco, 2000). In crabapple (*Malus hupehensis*), *F. mosseae* infection was also found to be associated with the upregulation of phosphorus starvation factors under phosphorus-deficient stress (Huang et al., 2025). In another recent study, the AMF *Claroideoglomus etunicatum* and *Acaulospora scrobiculata* significantly increased levels of both soluble and exchangeable calcium (Ca) in the rhizosphere soil of apple seedlings (*M. robusta*) cultivated in sterile substrate, relative to non-mycorrhizal plants. Additionally, inoculation with *A. scrobiculata* led to significantly higher expression of genes involved in Ca signaling in the plant roots (relative to non-mycorrhizal plants) (Fu et al., 2023). In a different study, micropropagated apple rootstock seedlings (M9T337) were inoculated with *Paraglomus* sp. in sterilized substrate. Although mycorrhizal colonization rates were not reported, AMF-inoculated plants were associated with significantly higher root, stem and leaf content of plant available nitrogen (NH_4_^+^and NO_2_^-^), relative to non-*Paraglomus* treatments (which received sterilized AMF as inoculum). AMF-inoculation also led to increased activity of key enzymes involved in the nitrogen cycle (in roots and leaves) as well as upregulation of nitrate and ammonium transporter genes in seedling roots (Wang et al., 2024a).

Further, in several studies, colonization by AMF (esp. *Rhizophagus irregularis*) has been shown to enhance drought tolerance in apple (Runjin, 1989; Huang et al., 2020, Huang et al., 2021a). Strigolactones, plant signaling molecules released from roots which trigger hyphal branching/encourage penetration by the fungi, appear to be an important aspect of this process (Akiyama et al., 2005; Huang et al., 2021a, Huang et al., 2021b; Xu et al., 2023). AMF-mediated drought tolerance in apple has also been found to involve the upregulation of drought-responsive genes in both host and fungal tissues, as well as physiological and transcriptional adjustments associated with: improved photosynthetic performance, enhanced antioxidant enzyme activity, and osmotic regulation (Huang et al., 2020). While these studies provide key foundational data, studies which extend beyond the use of apple seedlings (which are highly heterozygous) or micropropagated plantlets in sterilized substrates are required for the translation of results to field applications. Utilization of AMF in combination with host plant genetics requires evaluation of AMF-mediated benefits in orchard soil with commercially available clonal rootstock material, akin to those planted in nursery and orchard systems.

Compared with AMF-mediated drought tolerance in apple, research regarding the role of AMF in the control of soilborne diseases is limited. Generally, when field investigations of the effects of mycorrhizal inoculations on soilborne disease have been conducted, they have taken the form of measuring plant growth without direct measurement of pathogen infection in root tissue (e.g., Wang et al., 2024b). Of particular concern in modern, high-density tree fruit cropping systems which utilize dwarfing and semi-dwarfing rootstocks, is replant disease. Apple replant disease, caused by a complex of soilborne pathogens, reduces productivity of new plantings at sites previously planted to apple or closely related tree fruit, particularly those within the family Rosaceae. A core group of pathogens including multiple species of fungi (*Rhizoctonia solani* and *Cylindrocarpon/Ilyonectria* spp.) and oomycetes (*Globisporangium*/*Pythium* and *Phytophthora* spp.) have been repeatedly implicated in the disease in major apple-growing regions around the world (Mazzola, 1997; Somera and Mazzola, 2022). These pathogens typically act together with the root-lesion nematode *Pratylenchus penetrans*, which creates wounds that serve as entry points for fungal and oomycete pathogens. In one of the only studies investigating the interaction between AMF and *P. penetrans* infection in apple root tissue, Ottawa 3 apple rootstocks that had been pre-inoculated with either *F. mosseae* or *Rhizophagus intraradices* (prior to planting in the field) were shown to harbor fewer *P. penetrans* than non-inoculated control plants 2 years after planting into fumigated soil reinoculated with *P. penetrans* (Forge et al., 2001). The ability of apple to suppress root infection by replant pathogens depends, in part, on rootstock genotype (Mazzola et al., 2009; St. Laurent, et al., 2010) but little has been done to systematically assess apple rootstock sensitivity to individual components of the disease complex.

In a previous study by Cook et al. (2024), the ability of four different AMF species (*Rhizophagus irregularis, Septoglomus deserticola, Claroideoglomus claroideum* and *Claroideoglomus etunicatum*) to rapidly colonize a variety of commercially-available Geneva apple rootstock genotypes (G.11, G.41, G.210, G.969, and G.890) was tested. Among the AMF tested, *C. etunicatum* and *R. irregularis* were identified as the most “compatible” fungal partners, regardless of rootstock genotype. Although this study addressed rootstock/AMF species preferences, colonization by AMF could not be interpreted as directly functioning in a plant beneficial role. Therefore, in the present study, we aimed to assess several benefits of specific apple rootstock/AMF associations previously identified as compatible including: plant nitrogen uptake, tolerance to water stress and protection against root-pathogenic fungi. At the same time, we sought to better understand how certain context-dependent factors, such as host genotype, abiotic stress and/or soil microbial context influenced anticipated functional benefits. Specifically, the ability of *R. irregularis* to improve nitrogen uptake and tolerance to water stress in the dwarfing rootstock G.11 (with and without replant pressure) was tested (Experiment 1). A separate experiment (Experiment 2) tested the ability of two different AMF species (*C. etunicatum* and *C. claroideum*) to enhance plant defense against infection by the fungal replant pathogen *Rhizoctonia solani* anastomosis group (AG)-5 in G.41 and G.890. Together, these experiments provide new insight into specific apple rootstock/AMF associations with the potential to improve disease control, drought tolerance and sustainability in nursery and orchard systems.

## 2 Materials and methods

### 2.1 Experiment 1

Experiment 1 tested the impact of *R. irregularis* inoculation on the ability of G.11 apple rootstock to take up water and nitrogen from soil when experiencing water-stress and/or replant pressure.

#### 2.1.1 Planting materials

Experiment 1 utilized the dwarfing apple rootstock G.11, selected for its relatively small root system which increases susceptibility to water deficits (Casagrande Biasuz and Kalcsits, 2022). Rootstock plants (6.35 mm diameter) were sourced from Cameron Nursery (Eltopia, WA, USA) and grown in 2.7 L pots containing soil that was collected from WSU-Sunrise Research Orchard [47.311551, −120.068531] near Rock Island, WA in June 2024. This location is known to possess the consortia of soilborne pathogens defined as causal agents of apple replant disease (Mazzola, 1998). In this experiment, soil was either untreated (live) or pasteurized. Soil pasteurization was performed by heating moistened soil in a pasteurization oven at 80 °C for 12-18h on two successive days. Between heating sessions, soil was allowed to cool and was hand-mixed (without opening the bag) to break up any remaining soil chunks. Before planting, rootstocks were topped to remove apical tips, and fresh weight, and root volume (via water displacement) were measured. Initial mean trunk diameter (marked ~5 cm above the soil line) was measured at planting using a digital caliper at two perpendicular directions.

The AMF inoculant used in this experiment was *Rhizophagus irregularis* (MycoInTech, Tarragona, Spain, 217 spores/g), a species which was previously shown to rapidly form strong associations with Geneva apple rootstocks including G.11 (Cook et al., 2024). Prior to planting, 12 g of inoculant was applied directly to the root ball/planting hole to ensure propagule/spore contact. Pots were then backfilled with either live or pasteurized orchard soil and subsequently watered to remove air pockets.

#### 2.1.2 Experimental design

Experiment 1 included 2 different soil types (pasteurized vs. replant/live), 2 watering regimes (well-watered vs. water-stressed), and 2 inoculation levels (pre-inoculated with the AMF *R. irregularis* vs. uninoculated). These 8 different treatment combinations included: 1) Live orchard soil x AMF x WW (well-watered), 2) Live orchard soil x AMF x WS (water-stressed), 3) Pasteurized orchard soil x AMF x WW, 4) Pasteurized orchard soil x AMF x WS, 5) Live x No AMF x WW, 6) Live x No AMF x WS, 7) Past x No AMF x WW, and 8) Past x No AMF x WS. Each treatment had 7 biological replicates arranged in a block design, with one plant per treatment positioned randomly within each of 7 blocks. An additional block contained *R. irregularis*-inoculated and uninoculated G.11 rootstocks grown in either live or pasteurized soil (3 replicates each). These 12 “sentinel” plants were intentionally harvested 6 weeks after planting to check for AMF root colonization prior to the start of water stress.

All plants were grown in a temperature-controlled greenhouse (USDA-ARS, Wenatchee, WA, USA) at 21–29 °C. Supplemental lighting provided a 50:50 red:blue ratio with a 14 h photoperiod, augmenting natural light. Mean PPFD was 460 μmol/m²/s at canopy level, measured with an LI-180 instrument (LI-COR, Lincoln, NE, USA). Soil moisture and temperature were monitored using 5TM sensors (Campbell Scientific, Logan, UT, USA) inserted into the soil near the root zone in 3 replicates per treatment group. Data was logged every 30 minutes with Em50G data loggers (METER, Pullman, WA, USA).

#### 2.1.3 Confirmation of AMF colonization (6 weeks post-planting)

*R. irregularis* colonization of G.11 rootstocks was assessed 6 weeks after planting by harvesting sentinel plants. This was considered to be a relevant timeframe for sufficient colonization to occur (Reich, 1988; Cook et al., 2024). As noted above, three trees from each of four groups (inoculated live soil, uninoculated live soil, inoculated pasteurized soil, and uninoculated pasteurized soil), were destructively harvested. Plants were removed from their pots and loose/adhering soil was removed. Fine, new white root tissue was then sampled from various places on the root system and temporarily stored at 4 °C until further processing. The percentage of root colonization by AMF was assessed via root staining with trypan blue using a modified protocol by (Phillips and Hayman, 1970), as adapted by INVAM. Briefly, roots were rinsed clean in DI water and cut into ~ 2 cm segments before being placed in plastic tissue biopsy cassettes (Micro Mesh, Ted Pella, Inc., Redding, CA, USA). They were then cleared by immersion in a 10% (w/v) KOH solution for 90 minutes in a 95 °C water bath. Following clearing, cassettes were rinsed (≥ 5 changes of DI water) and submerged in a 2% (v/v) HCl solution for 1 hour at room temperature. Roots were then rinsed again and stained for 1 hour in a solution of 0.05% trypan blue in lactoglycerol (1:1:1, v:v:v; DI water, glycerol, lactic acid) in a 95 °C water bath. After staining, cassettes were thoroughly rinsed to remove excess stain (≥ 5 changes of DI water) and stored in DI water at 4 °C prior to microscopic examination.

To quantify the degree of mycorrhization, the gridline intersect method was employed (McGonigle et al., 1990). Each sample was analyzed separately with three technical replicate slides per plant. Root segments were arranged lengthwise in four rows on a microscope slide, mounted with polyvinyl-lactoglycerol (PVLG), and covered with a cover slip, gently flattening the roots. AMF structures (including hyphae, arbuscules, and vesicles) were recorded at each field of view using a compound microscope (Olympus BX53, Olympus Bartlett, TN, USA) at 40x magnification, examining >100 fields of view per slide. The percentage of AMF colonization was calculated as:


$$\begin{array}{l} \mathrm{\% AMF colonization}\\ =(\frac{\mathrm{number of fields of view with AMF structures}}{\mathrm{total number of fields of view}})\times 100 \end{array}$$

#### 2.1.4 Water stress conditions and labelled N spiking

During the initial six-week growing period, daily water usage for each plant was quantified. Pots were saturated to water runoff, allowed to drain for two hours, and then weighed to determine water holding capacity. Pots were weighed again, approximately 24 hours later, to calculate daily water usage (g/hour). During the water stress portion of the experiment (6–10 weeks post planting), WS plants were gradually stressed by providing 40-50% of their calculated water usage since the last watering (plants were watered every 48-hours or as needed). Adjustments to the amount of water provided were made based on results from the Ψ_stem_ (stem water potential) physiological test (detailed below) during this four-week period. WW plants received 100% of the calculated water usage since the last watering.

Upon initiation of the water stress portion of the experiment, ammonium-15N nitrate (^15^NH_4_NO_3_) was also spiked into experimental plants to track nitrogen uptake. Ammonium-nitrate, an inorganic nitrate salt, is widely used as a nitrogen fertilizer in agriculture. For each pot in blocks 1-6, 100 mg of ^15^NH_4_NO_3_ (10 atom % ^15^N) was dissolved in 10 ml distilled water in a 15 ml falcon tube, vortexed, and added to the plant’s water. For block 7 plants, unlabeled NH_4_NO_3_ was similarly prepared as a control. The ^15^NH_4_NO_3_ (or NH_4_NO_3_ control) solution was mixed into the plant’s assigned water volume (adjusting for the volume of the N solution) using a separate glass beaker to avoid cross-contamination. Plants were watered carefully to ensure any runoff containing labeled nitrogen was retained in aluminum dishes beneath each pot (**Figures 1A, B**). Separate utensils and beakers were used for labeled and unlabeled solutions, and work surfaces were covered during labeled nitrogen preparation to minimize contamination risk.

**Figure 1:**
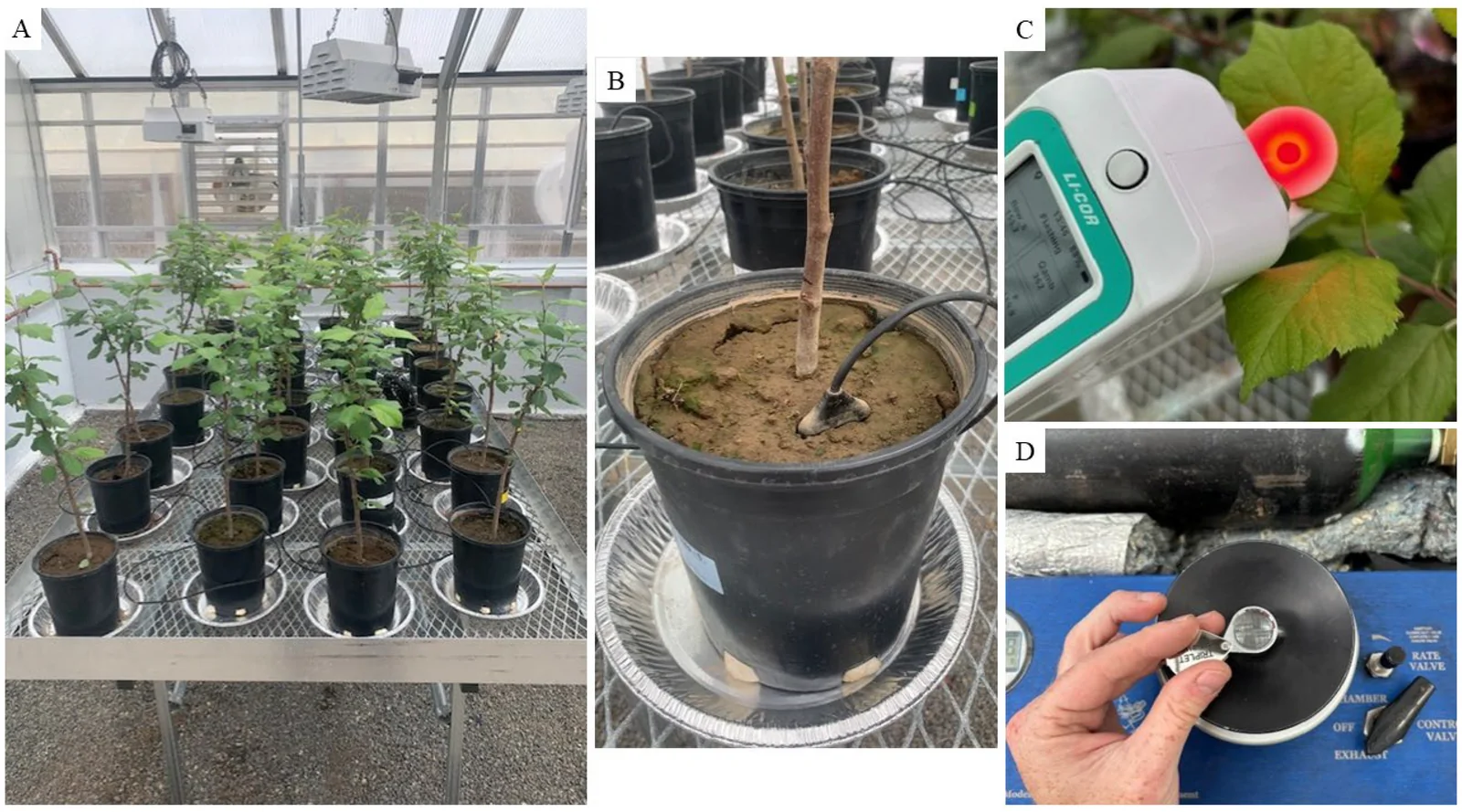
**(A)** Experiment designed to assess the ability of *R. irregularis* to improve tolerance to water stress in G.11. **(B)** G.11 rootstock in orchard soil with soil moisture sensor **(C)** Handheld device used to measure stomatal conductance non-destructively **(D)** Measuring Ψstem using a Scholander pressure chamber.

#### 2.1.5 Plant water relations

To assess plant water status/stress, both semi-destructive and non-destructive measurements were employed. The semi-destructive method involved measuring Ψstem (stem water potential) using a Scholander pressure chamber (Model 615D, PMS Instrument Company, Albany, OR, USA) (**Figure 1D**), following the protocol described by McCutchan and Shackel (2019). Briefly, mature, healthy leaves near the trunk were enclosed in black polyethylene bags and aluminum foil two hours prior to measurement. Ψstem was measured on one leaf per tree, once per week, at solar noon. The target value for Ψstem was < -1.0 MPa and watering was adjusted as needed if Ψstem was above this value. As mentioned above, WS pots were rehydrated to 50% of their water usage with adjustments based on weekly stem water potential measurements targeting –1 MPa (plants were rehydrated to 40% their water usage if above –1 MPa). WW pots were rehydrated to full saturation. The non-destructive method assessed stomatal conductance using mature, healthy, sun-facing leaves (like those used for Ψstem measurements). A LI-600 leaf porometer/fluorometer device was used (LI-COR, Lincoln, NE, USA) to measure stomatal conductance (**Figure 1C**). Stomatal conductance was recorded daily, alongside pot measurements, at solar noon, when minimum volumetric soil water content and peak plant transpiration rates would be expected.

#### 2.1.6 Plant harvest

The day before harvest, trunk diameter was measured at the same location as at planting (5 cm above the soil line). Leader shoot length was also recorded to quantify vertical vegetative growth over the experimental period. At 10 weeks post-planting, trees were destructively harvested by removing them from their pots and eliminating as much loose/adhering soil as possible. Fresh weight of the entire plant was measured, and root volume was determined using water displacement in a 2 L graduated cylinder. A subsample of fine, new white root tissue was then collected from each plant (7 biological replicates per treatment) and stored at 4 °C for percentage AMF colonization analysis (described above and provided in **Supplementary Data 2**) and at -80 °C for qPCR analysis (approximately 2.5 g of fresh root was removed from each plant in total). The whole tree was then dried in brown paper bags at room temperature for six weeks.

#### 2.1.7 Sample processing for ^15^N isotope analysis

Prior to labeled nitrogen analysis, the dry weight of each plant was recorded. Dried plant material was then separated into three compartments: roots, shoots, and leaves, with each sample processed individually. Roots and shoots were initially ground using a Wiley Mini-Mill (Thomas Scientific, Swedesboro, NJ, USA) to a particulate size of 425 microns, followed by further homogenization to a fine powder using a bead mill homogenizer (Powergen High Throughput Homogenizer, Fisher Scientific, Waltham, MA, USA). Leaves were directly homogenized into a fine powder using the bead mill homogenizer. Once processed to a fine powder, 3 mg of leaf and root samples and 6 mg of shoot tissues were weighed using a high-precision analytical balance (XSE105 DualRange, Mettler Toledo, Greifensee, Switzerland) and placed into 4 mm × 6 mm tin capsules (Costech Analytical, Valencia, CA, USA). The prepared capsules were then shipped to the UC Davis Stable Isotope Facility (Davis, CA, USA) for ^15^N isotope content analysis. The raw data from UC Davis (**Supplementary Data 1**) was then used to calculate 15N abundance for each plant tissue compartment using the formulas below with reference to Lan et al. (2024):


$$\text{Total amount N }(\text{mg})=\frac{\%\text{N}}{100}\text{ x dry weight }(\text{g})\text{ x }1000\text{ mg}/\text{g}$$

$$\begin{array}{c} \text{Ndff}(\%)\\ =(\frac{\text{Atom}\%\;15\text{N in sample }-\text{Atom}\%\;15\text{N in unlabeled control}}{\text{Atom}\%\;15\text{N for fertilizer }-\text{Atom}\%\;15\text{N in unlabeled control}})\;\times 100\% \end{array}$$

15N uptake =Ndff% x  total amount N (mg)

where dry weight for each plant compartment (root, wood, leaf) was estimated using apple-specific biomass partitioning (Valverdi et al., 2019) in which total plant biomass (g) was multiplied by 0.103, 0.53 and 0.367 to estimate root, wood, and leaf biomass, respectively. Ndff% represents the proportion of ^15^N absorbed by the plant (or plant compartment) that came from the labeled ^15^NH_4_NO_3_ fertilizer and ^15^N uptake represents the amount (mg) of ^15^N from fertilizer taken up by the plant compartment.

#### 2.1.8 DNA extraction from root tissue and qPCR analysis

Frozen root tissue was surface-sterilized by rinsing in sterile water to remove soil debris, followed by a 90-second soak in 6% bleach, and a final rinse with three changes of sterile DI water. Immediately after sterilization, 2 g of fine root tissue (roots < 1.5 mm in diameter) was selected from various locations within the sample to obtain a representative portion and cut into ~1 cm lengths. The samples were ground in a sterile mortar and pestle with liquid nitrogen to a fine powder and stored at -80 °C. A new sterile mortar and pestle was used for each experimental treatment.

DNA was extracted from 50 mg of the ground root tissue using the DNeasy Plant Pro Kit (Qiagen, Valencia, CA, USA), following the manufacturer’s instructions. Tissue disruption was performed using a FastPrep-96 high-speed homogenizer (MP Biomedicals, Irvine, CA, USA). The relative abundance of specific fungal and oomycete pathogens known to be part of the replant disease complex in Washington State were assessed via real-time qPCR using the Applied Biosystems QuantStudio 3 system (Foster City, CA, USA). *Cylindrocarpon (Ilyonectria)* spp. DNA was quantified using the primer pair YT2F (5’-GAT GAA GAA CGC AGC GAA AT-3’) and CylR (5’-TGT GCT ACT ACG CAG AGG AA-3’), with reaction mixtures and amplification conditions described by Tewoldemedhin et al. (2011). A standard curve was prepared from genomic DNA of *I. robusta* isolate 14-264 (100–0.01 pg µl^-^¹). *Rhizoctonia solani* DNA was quantified using the primer pair Rhsp1 (5’-AAC AAG GTT TCC GTA GGT G-3’) and ITS4B (5’-CAG GAG ACT TGT ACA CGG TCC AG-3’), following reaction conditions described by Bruns et al. (1991) and Gardes and Bruns (1993). The standard curve was prepared from genomic DNA of *R. solani* isolate 5-103 (100–0.01 pg µl^-^¹). *Globisporangium ultimum* DNA was quantified using the primer pair ULT1F (5’-GAC ACT GGA ACG GGA GTC AGC-3’) and ULT4R (5’-AAA GGA CTC GAC AGA TTC TCG ATC-3’), according to the reaction protocol described by Schroeder et al. (2006). The standard curve was prepared from genomic DNA of *G. ultimum* isolate 64-1276 (100–0.01 pg µl^-^¹). *Phytophthora cactorum* DNA was quantified using the primer pair Ypt cacF3 (5’-CAT GGC ATT ATC GTG GTG TA-3’) and Ypt cacR3 (5’-GCT CTT TTC CGT CGG C-3’), following amplification conditions described by Li et al. (2013). The standard curve was prepared from genomic DNA of *P. cactorum* isolate 60-1190 (1000–0.1 pg µl^-^¹). In addition to pathogen-specific qPCR assays, *R. irregularis* DNA was quantified relative to apple DNA in root tissue collected at harvest using the methods previously described in Heller and Carrara (2022) with 10% input volume (2.5ul DNA sample in 25 ul reaction volume), and apple-specific elongation factor 1α (EF1) primers and probe from Gusberti et al. (2012). This method was added subsequent to the initial root tissue collection (6-week post-planting) in which all of the sample was used for microscopic assessment of AMF. Thus, we were only able to assess samples collected at harvest in this way.

#### 2.1.9 Statistical analysis

All statistical analyses and visualizations were conducted using GraphPad Prism 10.4.2 (GraphPad Software, San Diego, CA, USA). For percent root length colonization, data was transformed (y=log(y)), and nested one-way ANOVA (with subcolumns representing triplicate measurements from each tree) was performed to compare treatment means. Homoscedasticity and normality of data was visually assessed via residual and QQ plots; pairwise differences were tested with Tukey’s multiple comparisons test. Statistical significance was defined as p < 0.05. Soil moisture data were subset by selecting daily measurements recorded at 1:00 p.m. for each sensor. Data from live and pasteurized soils were analyzed separately. For each timepoint, unpaired t-tests were used to compare soil moisture between WW and WS treatments, with and without AMF inoculation. For stomatal conductance, measurements were taken on the same set of 20 sampling dates (7 plants per treatment). Repeated measures (RM) one-way ANOVA (with Geisser-Greenhouse correction) was used to control for daily weather variations (measurements matched by day). Before conducting these tests, we checked for outliers (ROUT method) and normality (Shapiro-Wilk and Kolmogorov-Smirnov tests) across treatment groups. To test the effects of AMF inoculation on stomatal conductance, plants experiencing the same soil type and watering regime were compared (Šidák’s multiple comparison tests). To investigate the impact of soil type (live vs. pasteurized) on stomatal conductance, plants with same watering regime x AMF status were compared (Šidák’s multiple comparison tests). For Ψstem, a one-way ANOVA was used to test differences among treatments, and the Brown-Forsythe test was applied to assess homogeneity of variances. Tukey’s test was used for pairwise comparisons. Within each soil type, the effect of treatment on total ^15^N fertilizer recovery and partitioning of ^15^N into roots vs. shoots was tested with a non-parametric Kruskal-Wallis test and Dunn’s multiple comparison tests for *post hoc* analyses.

Two-way ANOVA was used to determine how pathogen root density was affected by soil type (live vs. pasteurized) and treatment (water regime x AMF status). For both *R. solani* and *Cylindrocarpon (Ilyonectria)* spp., the effect of soil type was highly significant (p< 0.01). Šidák’s multiple comparisons test was used to test for significant differences in pathogen levels between live vs. pasteurized soil for plants experiencing the same watering regime x AMF status.

Growth parameters (e.g., trunk diameter, leader shoot length) were analyzed descriptively, with means and standard deviations calculated for each treatment. Normality was assessed as described above. For non-normally distributed data (root volume/trunk diameter), a non-parametric Kruskal-Wallis test followed by Dunns multiple comparisons were used to compare group means. When the data was normally distributed (fresh weight/leader shoot length), a one-way ANOVA followed by Tukey’s multiple comparisons test was used to compare group means.

### 2.2 Experiment 2

Experiment 2 evaluated the ability of select AMF x apple rootstock associations to suppress infection by the fungal replant pathogen *Rhizoctonia solani* AG-5, an anastomosis group which has been shown to be highly virulent towards apple (Mazzola, 1997).

#### 2.2.1 Initial planting/inoculation with AMF

One-year-old tissue-cultured apple Geneva rootstock plantlets (G.41, G.890) were obtained from North American Plants, Inc. (McMinnville, OR, USA). *Claroideoglomus* species previously shown to form strong associations with apple (*C. claroideum* and *C. etunicatum*) were obtained from MycoInTech (Tarragona, Spain) as single-species AMF inoculants. To establish AMF symbiosis prior to infection with *R. solani*, 12 plants of each genotype were inoculated with 5 g (1 tsp) of either *C. claroideum*, *C. etunicatum*, or left uninoculated as controls. Inoculant was sprinkled onto the root ball/planting hole to maximize contact with roots. Due to insufficient inoculum (manufacturer unable to send the requested amount), the G.890 × *C. claroideum* treatment was excluded, resulting in a total of 5 treatments (60 plants).

Plant fresh mass was recorded prior to planting into 1 L pots containing pasteurized orchard soil (WSU-Sunrise Research Orchard, Rock Island, WA, USA). Plants were maintained in a walk-in growth chamber (Conviron, Pembina, ND, USA) under a 16 h photoperiod, 18–22 °C, and ~80 PPFD at canopy level. After 5 weeks, fine roots from three plants in each treatment were harvested, and AMF colonization was confirmed microscopically (**Supplementary Data 2**). Root processing and analysis followed the same procedure as described in Experiment 1.

#### 2.2.2 Preparation of pathogen inoculum

The pathogen used was a strain of *Rhizoctonia solani* AG-5 (lab isolate #5-104) recently passed through apple seedling roots and re-isolated. Inoculum was prepared on whole oat grains following a similar procedure as outlined in Mazzola, 1997. Erlenmeyer flasks (250 ml) were sterilized and fitted with cotton–cheesecloth stoppers. Each flask then received 50 ml oats and 15 ml DI water, was mixed, and left to soak for 2 hours. Moistened oats were autoclaved at 121 °C for 20 min, stored overnight in a biosafety hood, autoclaved a second time under the same conditions, and allowed to completely cool. One half of petri dish (90 mm diameter) containing *R. solani* AG-5 growing on PDA + ampicillin (100 µg/ml) was cut into 0.5 cm cubes and used to inoculate the flasks. The flasks were then shaken to evenly distribute inoculum, sealed with parafilm, and incubated at ambient temperature in the dark for 4 weeks. Infested oats were removed from the flasks under sterile conditions, dried overnight, homogenized in a bullet blender, and ground to remove large fragments. To confirm viable *R. solani* AG-5 infestation, the final ground oat material was plated on PDA + ampicillin (100 µg/ml) and *R. solani* was verified morphologically by bright-field microscopy (**Supplementary Figure 1**). Infested soil was produced by hand-mixing 2.7 L of pasteurized orchard soil with 6.25 g of *R. solani*-infested ground oats (0.25% w/w soil ratio) in a clean bin for 1–2 min to ensure even distribution.

#### 2.2.3 Experimental planting (6 weeks post-AMF inoculation)

6 weeks after planting into pasteurized orchard soil, AMF-colonized plants were removed from their pots, roots gently agitated to remove loose adhering soil, and fresh weight recorded. Mycorrhizal and uninoculated control plants were then transplanted into 2.7 L pots containing *R. solani* AG-5-infested pasteurized orchard soil (7 replicates per each of the 5 treatments). Plants were then returned to the growth chamber under the conditions described above and arranged in a randomized complete block design, with one per plant per treatment positioned randomly within each of 7 blocks. Pots were placed in aluminum dishes to prevent cross-contamination of AMF spores or pathogen inoculum with neighboring plants. Plants were hand watered every 48 hours or as needed. Due to growth chamber breakdown shortly after transplantation, plants were moved into the greenhouse where they remained for the duration of the experiment under the same conditions described in Experiment 1.

#### 2.2.4 Experiment harvest

All plants were destructively harvested 4 weeks after planting into *R. solani* AG-5-infested pasteurized orchard soil (10 weeks after the initial planting). The pots were overturned, roots freed by gentle shaking, and rhizosphere soil collected and stored on ice. Plant fresh weight was recorded, and fine, white root tissue was excised with ethanol-cleaned snippers and partitioned into two sample bags (for staining/microscopy vs. qPCR). All samples were kept on ice during harvest; root tissue for qPCR was frozen at –80 °C and roots for microscopy were stored at 4 °C.

#### 2.2.5 Processing of root tissue and qPCR

Root tissue for microscopy was processed, stained, and percentage AMF colonization calculated per the methodology described in Experiment 1. Frozen root tissue was surface sterilized and ground in liquid nitrogen prior to DNA extraction as described in Experiment 1. Relative quantities of *R. solani* AG-5 DNA were assessed by real-time qPCR using the primer pair RsAG5F (5’-GAT ATT TGT AGC TGG CTC ATG-3’) and RsAG5R (5’-GCA CCA ATT GTT CTT AAA AAA CAA TC-3’) (Mazzola and Zhao, 2010). All reactions were run on an Applied Biosystems QuantStudio 3 system (Foster City, CA, USA) under the conditions previously described by (Mazzola and Zhao, 2010). A standard curve was generated from purified DNA of *R. solani* AG-5 (isolate #5-104), diluted from 100 pg µl^-^¹ to 0.01 pg µl^-^¹.

#### 2.2.6 Statistical analysis

Quantitative PCR (qPCR) data for *R. solani* AG-5 quantity in apple root tissue were normalized to pg DNA per ng input DNA to correct for variation in DNA extraction efficiency. Statistical analysis of G.41 data was performed via one-way ANOVA on ln transformed values followed by Dunnett’s multiple comparisons test; data for G.890 was normal/non-transformed and was analyzed via an unpaired t test with Welch’s correction.

## 3 Results

### 3.1 Experiment 1: Assessing the effects of *R. irregularis* inoculation on tolerance to water stress in G.11

“Sentinel” plants were harvested 6-weeks after planting to assess AMF colonization levels in root tissue. Microscopic assessment indicated that AMF were actively colonizing G.11 rootstocks cultivated in both live and pasteurized soil containing the *R. irregularis* inoculum; mean AMF root length colonization was 25 and 33% in live and pasteurized soil, respectively (**Figure 2**). In comparison, uninoculated control plants appeared to have little to no AMF colonization at this time. At harvest (10 weeks post-planting), mean percent colonization ranged from 5-29% and 16-30% in live and pasteurized soil, respectively (**Figure 2**). No significant differences in percent colonization were identified between any of the pasteurized treatments. In live soil, however, percent colonization in the AMF x WW treatment was significantly higher than both WS treatments. It also is interesting to note that mean colonization rates were always higher in WW compared to matching WS treatments (albeit not always significant). This was also true of AMF vs. No AMF treatments experiencing the same conditions (**Figure 2**). It is also worth noting that AMF colonization was generally lower in live (relative to pasteurized) soil when comparing otherwise identical treatments (**Figure 2**).

**Figure 2:**
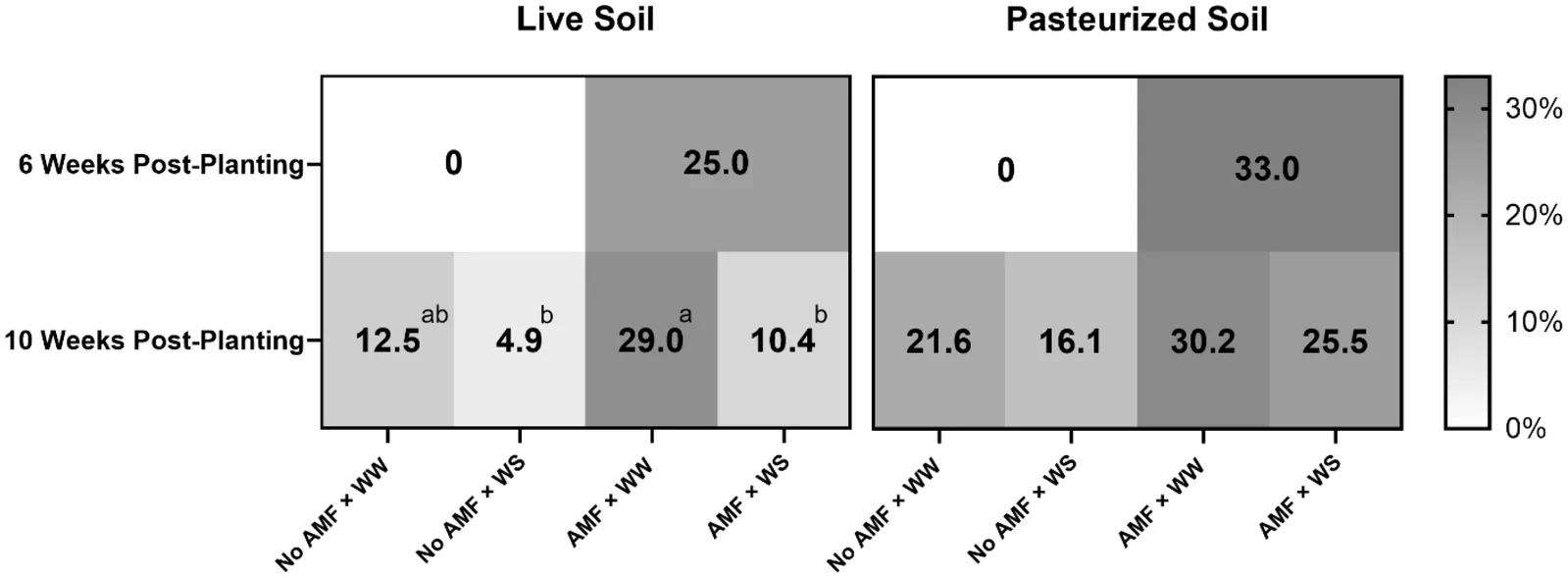
Average AMF root length colonization in fine roots of G.11 rootstocks grown in either live (left) or pasteurized (right) soil. Root length colonization was measured at two timepoints: 6 weeks post-planting into soil with or without the *R. irregularis* inoculant and 10 weeks post-planting (i.e., 4 weeks after the application of water stress/labeled nitrogen); water-stressed (WS), well-watered (WW). Shading indicates the percentage of colonization, with dark and white/light shades representing highest and lowest values, respectively. Within each soil type, statistically significant differences between treatments are represented by different letter groups (nested one-way ANOVA, Tukey’s multiple comparison tests, p < 0.05).

In addition to microscopic assessment, the presence of *R. irregularis* DNA in roots (and a variety of other AMF) were quantified via species-specific multiplex qPCR assays. At this time (10 weeks post-planting), no significant differences in the amount of *R. irregularis* DNA were identified between any of the treatments (**Supplementary Figure 6**). Among several AMF species detected by the multiplex assays (incl. *Gigaspora* spp., *Rhizophagus intraradices*, *Claroideoglomus claroideum*, *C. etunicatum, Septoglomus constrictum* and *Funneliformis mosseae*), *R. irregularis* was the primary AMF DNA detected in root tissue, regardless of treatment (49/55 samples). Contributions from native/pre-existing *R. irregularis* strains cannot, however, be separated from that of the inoculated strain. Finally, it is also of interest that significantly more (46X) *R. intraradices* DNA was detected in WS than in WW plants cultivated in live x uninoculated soil (**Supplementary Figure 7**). *R. intraradices* was not, however, detected in live x *R. irregularis*-inoculated treatments, possibly indicating that the applied *R. irregularis* reduced native colonization by *R. intraradices*.

Volumetric soil water content was lower in the root zones of water-stressed plants than those of well-watered plants, regardless of inoculation or soil pasteurization status (**Supplementary Figure 2**). In general, differences between WW and WS soils widened over time, with volumetric soil water content showing a gradual decrease over time in WS treatments. This reflects our experimental technique, which was to decrease overall soil moisture/induce water stress gradually (i.e., WS soils were provided 40-50% of their water usage since the last watering, while WW soils were returned to full saturation). This data also mirrors pot weights, which remained stable in WW plants but declined gradually in WS plants (**Supplementary Figure 3**).

Regardless of soil type (live vs. pasteurized), WW plants always had significantly higher (less negative) Ψstem than WS plants (p < 0.0001), confirming greater stress in the latter. No significant differences in Ψstem were observed between inoculated vs. uninoculated plants with the same water-regime/soil type. As expected, G.11 rootstocks cultivated in WS conditions with a 50-60% water deficit from their calculated daily water usage need, had reduced stomatal conductance relative to those cultivated in WW soil (**Figure 3**). In live orchard soil, *R. irregularis* had a significant, positive effect on stomatal conductance under both well-watered and water-stressed conditions (**Figure 3A**; RM one-way ANOVA followed by Šidák’s multiple comparisons test: p =0.0019 and p = 0.0002, respectively). In pasteurized soil, when plants were well-watered, pre-colonization by *R. irregularis* resulted in reduced stomatal conductance relative to uninoculated plants (**Figure 3B**; RM one-way ANOVA followed by Šidák’s multiple comparisons test: p=0.02).

**Figure 3:**
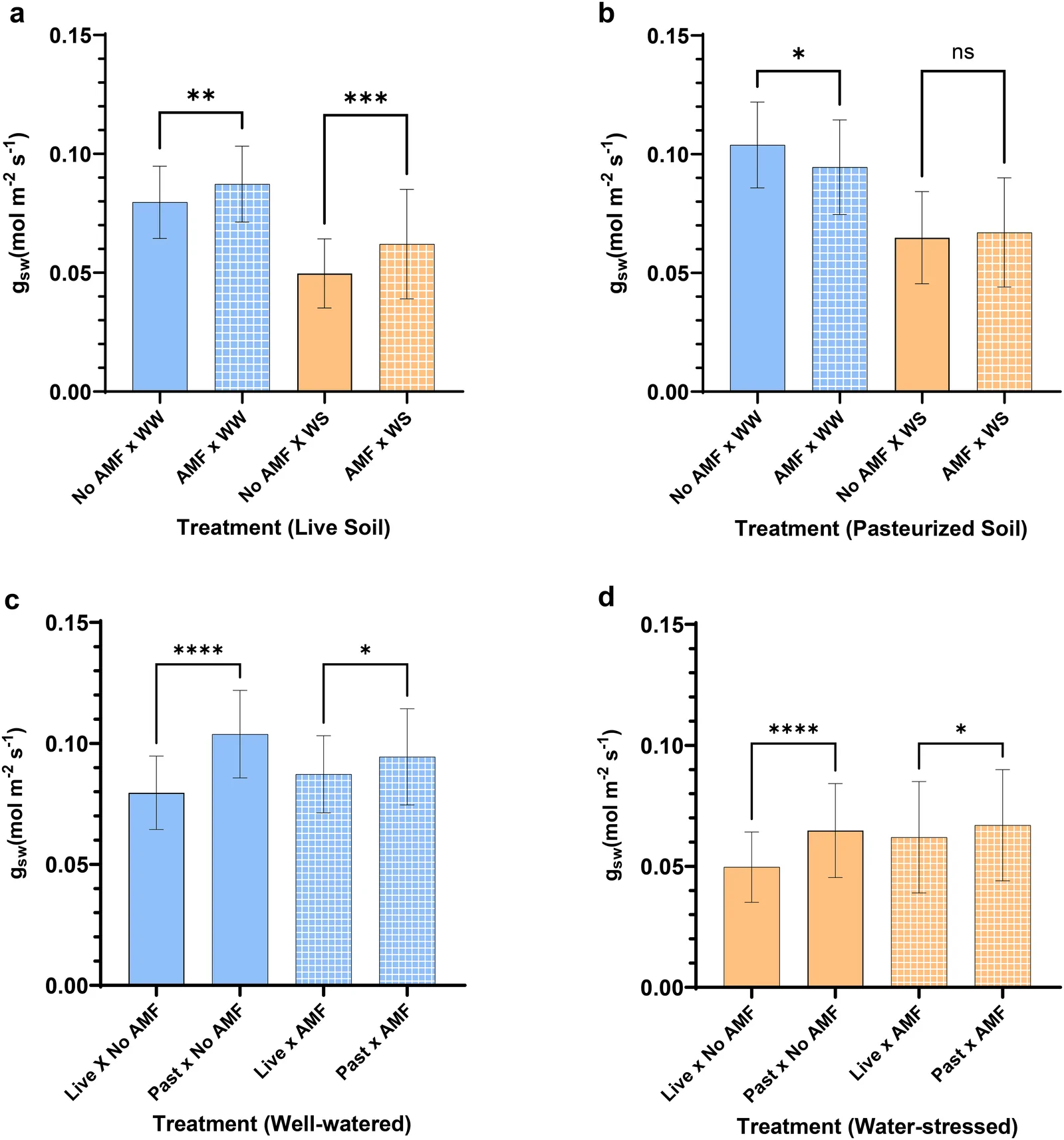
The effect of AMF inoculation on stomatal conductance by G.11 rootstocks cultivated in live **(A)** or pasteurized **(B)** orchard soil with (WS; orange bars); or without (WW; blue bars) a water deficit. Hatched bars represent treatments in which plants were colonized by *R. irregularis* prior to experiencing the different watering regimes. The impact of soil type (live vs. pasteurized) on stomatal conductance under WW **(C)** or WS **(D)** conditions was also assessed. Measurements were taken on the same set of 20 sampling dates (7 plants per treatment). Repeated measures one-way ANOVA was used to control for daily weather variations (measurements matched by day); Šidák’s multiple comparisons test was used to compare the means of pre-selected columns. Asterisks represent p-value thresholds: *(p<0.05), **(p<0.01), ***(p<0.001), ****(p<0.0001), ns, not significant.

For plants with the same watering regime x AMF status, stomatal conductance was, however, always significantly reduced under replant conditions (**Figures 3B, C**). The abundance of several replant pathogens (*Cylindrocarpon/Ilyonectria* spp., *Rhizoctonia solani*, *Globisporangium ultimum*, and *Phytophthora cactorum*) in G.11 root tissue were quantified using pathogen-specific qPCR assays. Neither *G. ultimum* nor *P. cactorum* were detected in any root samples. However, the abundance of both *Cylindrocarpon (Ilyonectria)* spp. and *R. solani* detected in roots was always significantly higher when plants were cultivated in live vs. pasteurized soil, regardless of treatment (WW x No AMF, WW x AMF, WS x No AMF, WS x AMF; Two-way ANOVA followed by Šidák’s multiple comparisons test; p<0.01 and p < 0.003 for all *Cylindrocarpon (Ilyonectria)* spp. and *R. solani* comparisons, respectively, **Supplementary Figure 8**). On the other hand, pathogen levels were not significantly different between AMF vs. No AMF treatments, regardless of watering regime.

### 3.2 Experiment 1: Assessing the effects of *R. irregularis* inoculation on plant nitrogen uptake in G.11

As part of the same experiment, we tested the ability of *R. irregularis* to improve nitrogen uptake in G.11. In the absence of replant pressure (pasteurized soil), ^15^N recovery between AMF and non-AMF plants was not significantly different, regardless of watering regime (**Figure 4B**). However, when cultivated in live soil and well-watered, significantly more ^15^N enriched nitrogen (~42%) was taken up by plants pre-colonized by *R. irregularis* than uninoculated plants growing under the same conditions (**Figure 4A**; p=0.01). Under these conditions (live x AMF x WW), inoculated plants acquired almost twice as much ^15^N from soil. This amount (6 mg) was similar to the level of ^15^N acquired when plants were grown in the absence of replant pathogens (pasteurized x AMF x WW). In comparison, *R. irregularis* did not significantly affect ^15^N from fertilizer measured in live x water-stressed treatments (**Figure 4A**).

**Figure 4:**
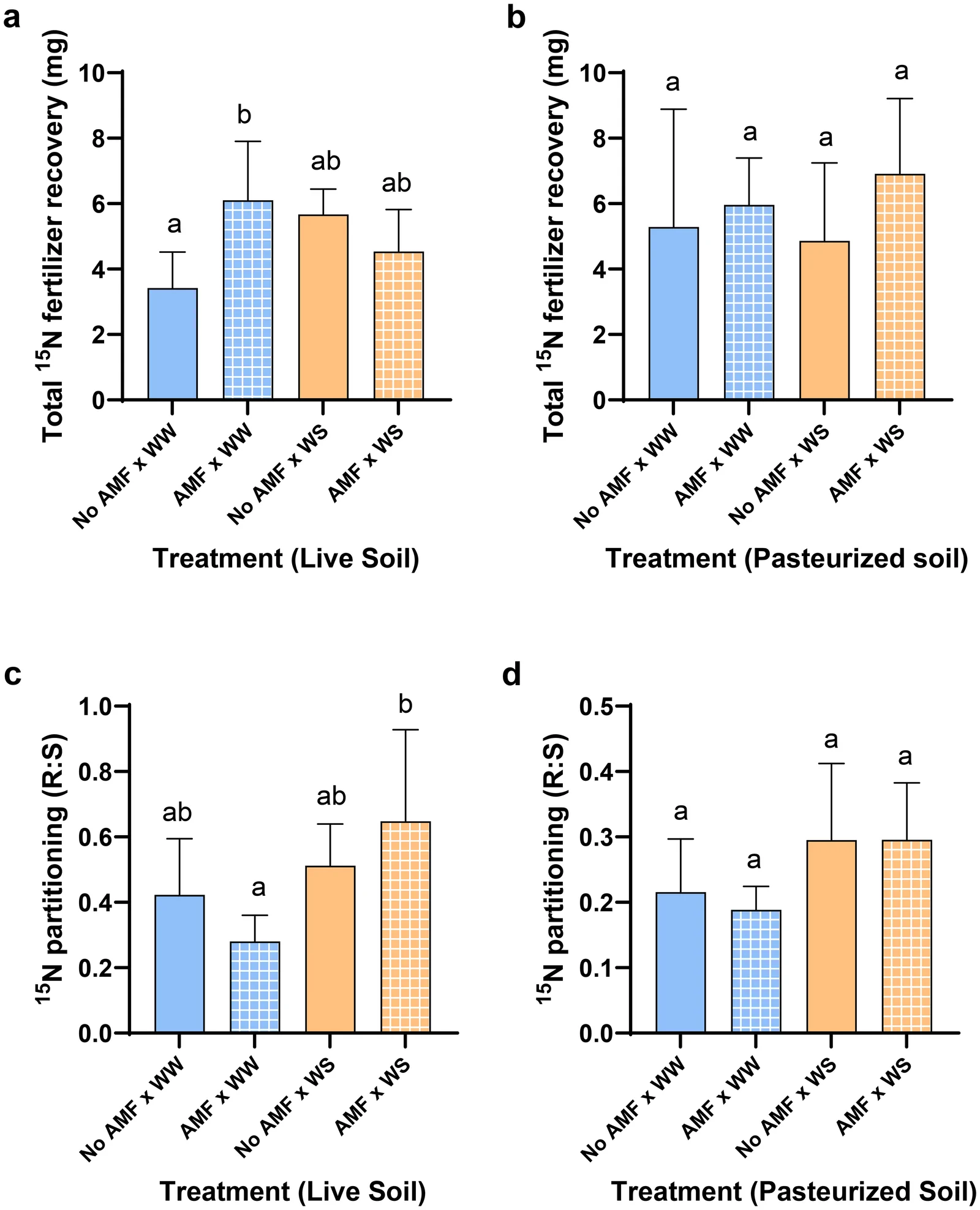
Bar graphs showing total ^15^N fertilizer recovery into G.11 rootstocks (roots + wood + leaves) cultivated in either live (replant) soil **(A)** or pasteurized soil **(B)** after 1 month. Partitioning of ^15^N recovered from ^15^N-enriched fertilizer into roots vs. shoots (R:S) in cultivated in live **(C)** and pasteurized **(D)** soil under different watering regimes. Well-watered (WW) treatments are shown in blue, while water-stressed (WS) treatments are colored orange. Hatched bars represent treatments in which plants were colonized by the AMF *R. irregularis* prior to experiencing the different watering regimes; n = 6 for all treatment combinations, except Live x AMF x WS where n = 5. Within each soil type, statistically significant differences between treatments are represented by different letter groups (Kruskal-Wallis test, Dunn’s multiple comparison tests, p < 0.05).

In terms of ^15^N partitioning between plant tissues, relative ^15^N enrichment followed a typical hierarchical pattern, with roots being the most enriched, followed by woody stems and leaves (**Supplementary Figure 5**). Water-stressed plants generally contained proportionally more labeled N in roots vs. shoots than well-watered plants, regardless of other treatment factors (**Figure 4C** and D). However, in live soil, water-stressed plants that had been pre-colonized by *R. irregularis* partitioned the most enriched nitrogen into their roots (**Figure 4C**). No significant differences in root:shoot partitioning of ^15^N were identified between pasteurized soil treatments (**Figure 4D**).

### 3.3 Experiment 1: Growth

While the primary aim of this study was to test the ability of AMF to improve plant nutritional and water status of compatible apple rootstock/AMF associations under agriculturally-relevant environmental conditions, plant growth data was also collected upon harvest. Overall, well-watered treatments were associated with significantly greater increases in fresh weight and root volume than water-stressed treatments (**Table 1**). However, no differences in total dry weight or leader shoot length were identified between any of the treatments. This result indicates that the degree of water-stress (imposed over the relatively short duration of the experiment) led to reduced tissue water content but did not significantly inhibit plant biomass accumulation. After 10 weeks, the amount of new trunk growth was also similar for most treatments. In pasteurized soil, however, the relative increase in trunk diameter in well-watered plants that had been pre-inoculated with *R. irregularis* (Past × AMF × WW) was significantly greater than water-stressed and uninoculated plants (Past x No AMF x WS).

**Table 1:** Effect of experimental treatment on average growth characteristics of G.11 rootstocks 10 weeks post-planting.

Watering regime	Soil	Inoculation status	Fresh weight change (g)	Total dry weight (g)	Leader shoot length (cm)	Root volume change (ml)	Trunk diameter change (mm)
WW	Pasteurized	Uninoculated	80.8 ± 37.0 ab	43.2 ± 4.6 a	14.9 ± 4.5 a	31.7 ± 14.7 ab	1.0 ± 0.9 ab
WW	Pasteurized	*+R. irregularis*	85.8 ± 17.9 a	39.7 ± 6.3 a	11.9 ± 2.8 a	54.3 ± 14.0 a	0.7 ± 0.3 a
WW	Live	Uninoculated	50.6 ± 10.8 bc	35.4 ± 4.5 a	11.8 ± 2.7 a	32.9 ± 7.6 ab	1.0 ± 1.4 ab
WW	Live	*+R. irregularis*	75.2 ± 24.0 ab	40.5 ± 6.7 a	16.7 ± 1.8 a	34.3 ± 9.8 ab	0.7 ± 0.4 ab
WS	Pasteurized	Uninoculated	26.3 ± 10.8 c	35.8 ± 5.1 a	12.1 ± 4.7 a	23.3 ± 8.2 ab	0.2 ± 0.1 b
WS	Pasteurized	*+R. irregularis*	31.0 ± 14.9 c	38.3 ± 11.6 a	12.3 ± 4.8 a	20.0 ± 12.7 b	0.6 ± 0.3 ab
WS	Live	Uninoculated	27.7 ± 9.0 c	38.5 ± 4.3 a	12.3 ± 2.5 a	17.1 ± 7.6 b	0.5 ± 0.5 ab
WS	Live	*+R. irregularis*	21.3 ± 11.4 c	35.7 ± 6.3 a	16.8 ± 5.7 a	15.0 ± 9.8 b	0.5 ± 0.3 ab

Number after ± is the standard deviation value. Fresh weight, root volume, and trunk diameter measurements were obtained at planting and also at harvest. Total plant dry weight was measured at harvest, following collection of a 2.5g subsample of root tissue from each plant. For normally distributed data (fresh weight change, total dry weight, and leader shoot length), one-way ANOVA followed by Tukey’s multiple comparisons test was used to compare group means. For non-normally distributed data (increase in root volume and trunk diameter), Kruskal-Wallis test followed by Dunn’s multiple comparison test was used. Significant differences within columns are represented by different letter groups. WW = well-watered and WS = water-stressed.

### 3.4 Experiment 2: Assessing the ability of select apple rootstock/AMF associations to limit infection by *Rhizoctonia solani* AG-5

In a separate experiment, we assessed the ability of select apple rootstock/AMF associations including: G.41 x *Clarideoglomus claroideum* G.41 x *Claroideoglomus etunicatum* and G.890 x *C. etunicatum* to reduce infection by the fungal replant pathogen *R. solani* AG-5. It was expected that specific apple rootstock/AMF combinations would differentially influence plant capacity to restrict root colonization by *R. solani* AG-5. After 5 weeks of growth in pasteurized orchard soil with AMF inoculant, mycorrhizal colonization was confirmed: average root length colonization was 13%, 18%, and 27% in G.41 x *C. claroideum*, G.41 x *C. etunicatum*, and G.890 x *C. etunicatum*, respectively. At this time, the degree of mycorrhization detected in uninoculated control plants was minimal (**Supplementary Data 2**).

Four weeks after transplantation into pasteurized orchard soil infested with *R. solani* AG-5, the amount of pathogen DNA present in the roots of G.41 plantlets pre-colonized by *C. etunicatum* was significantly reduced relative to uninoculated control plants (**Figure 5**). In G.890, pre-colonization by *C. etunicatum* did not reduce infection relative to controls (even though percent AMF colonization was higher in G.890 x *C. etunicatum* than in G.41 x *C. etunicatum* prior to transplantation). Although *C. claroideum* did not significantly reduce the amount of *R. solani* AG-5 present in G.41 relative to controls, pathogen quantity was lower, on average, in inoculated plants (**Figure 5**).

**Figure 5:**
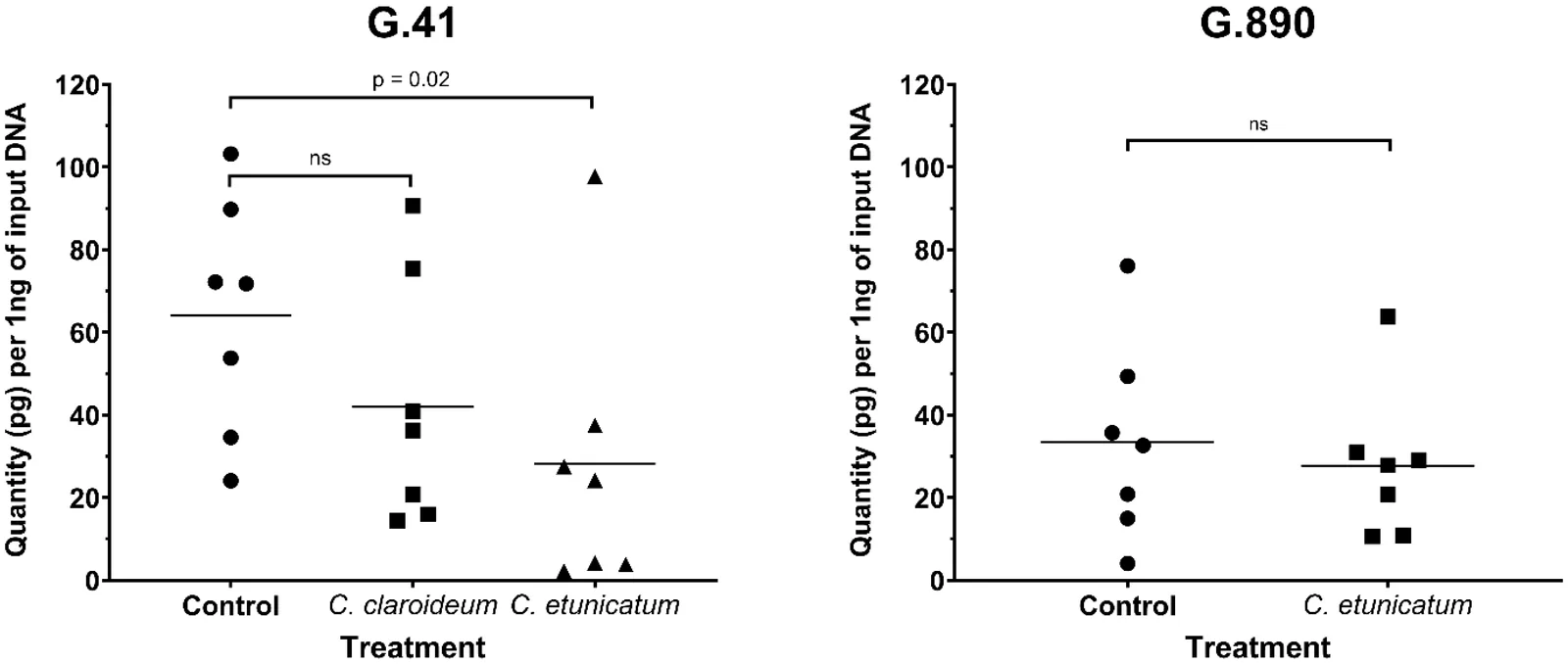
Amount of *R. solani* AG-5 DNA present in apple root tissue 4-weeks after transplantation into pasteurized orchard soil containing *R. solani* AG-5. Plants were either pre-colonized with the AMF *C.claroideum, C. etunicatum* or not colonized (Control) prior to transplantation. Each point represents the amount of *R.solani* AG-5 DNA present in a particular plant. Statistical analysis of G.41 data was performed via Ordinary 1-way ANOVA on ln transformed values followed by Dunnett’s multiple comparisons test; data for G.890 was normal/non-transformed and was analyzed via an unpaired t test with Welch’s correction.

## 4 Discussion

This study examined several key functional benefits of specific apple rootstock–AMF associations. Results provide clear evidence of AMF directly functioning in beneficial roles including: replant disease mitigation, drought stress alleviation, and plant nitrogen uptake and/or regulation of N distribution within the plant. These findings identify specific relationships that could be harnessed to improve the performance of plants experiencing a combination of stressors. In live (pathogen-infested) orchard replant soil, inoculation with the AMF *R. irregularis* significantly improved water-use efficiency in G.11 rootstocks under both water-stressed and well-watered conditions. The abundance of *Cylindrocarpon* (*Ilyonectria*) spp. and *R. solani* detected in roots was significantly higher when plants were cultivated in live vs. pasteurized soil, regardless of treatment. The results suggest that, regardless of external watering regime, inoculation with *R.irregularis* helped to alleviate the negative effects of root tissue damage caused by replant pathogens in live soil. Specific compensation mechanism(s) by which pre-inoculation with *R. irregularis* helped plants tolerate replant pressure (in live soil) are unknown. Replant pathogens do, however, inhibit plant absorption of water and nutrients by targeting fine roots. Therefore, growth of extraradical mycelium may have helped compensate for lost root tissue by directly contributing to the movement of water to the plant.

In addition, significantly more fertilizer-derived ^15^N was extracted from live soil in *R. irregularis-*inoculated vs. uninoculated plants under well-watered (but not water-stressed) conditions. Nitrogen uptake is regulated, in part, by the amount of water available in the soil (Plett et al., 2020). This may be one reason for the lack of significant differences between AMF and non-AMF plants experiencing water-stress, in conditions where there may have been less demand for N from soil. On the other hand, when a lack of water limits growth, plants may retain nitrogen in roots to maintain vital functions. Under combined water-stress and replant pressure (live soil), when the need to shift host resources from vegetative shoot growth to root growth would be even higher, pre-inoculation with *R. irregularis* enhanced the ability of G.11 to retain/accumulate nitrogen in root tissue.

These findings support previous studies documenting the capacity of *R. irregularis* to regulate water/nutrient relations in apple. However, as noted in the Introduction, most of these studies demonstrated the ability of AMF to mediate drought stress in apple seedlings when cultivated in *sterilized* substrate (Huang et al., 2020, Huang et al., 2021a, Huang et al., 2021b; Xu et al., 2023). In contrast, our study found there was a notable lack of AMF-mediated benefits (in terms of stomatal conductance or ^15^N recovery) in G.11 rootstocks cultivated in pasteurized soil. The reason for this result is not clear. Seedlings and rootstocks differ fundamentally in their root architecture, root age distribution, as well as root exudate chemistry, properties which may affect how the plant interacts with AMF. Additionally, plants experiencing fine root tip attrition resulting from replant pathogen attack are expected to be more dependent on AMF for mobilizing water/nutrients than plants with intact fibrous roots (grown in pasteurized soil). Orchard soil fumigation is a pre-plant treatment used to control replant disease; however, disease-control benefits are limited to approximately 1 year. After that, orchard soil re-establishes a microbial community that is indistinguishable from that found in the corresponding unfumigated replant soil (Mazzola et al., 2015; Wang and Mazzola, 2019). The results of this study emphasize the potential importance of AMF in alleviating plant water/nutrient stress, particularly under chronic disease state field conditions.

Along these same lines, a significantly greater amount of fertilizer-derived ^15^N was recovered by *R. irregularis* inoculated vs. uninoculated plants when well-watered and cultivated in live (replant-conducive) soil, an effect that was absent in well-watered pasteurized soil. Thus, in the present study, AMF-mediated benefits appeared to depend on pre-existing microbial context, a finding mirrored across several cropping systems (Lutz et al., 2023; Cucu et al., 2026). The possible involvement of other (beneficial) microorganisms as interacting players in live soil treatments should also be mentioned. This is an actively growing area of research, and several studies have documented the participation of both AMF and bacteria in providing specific benefits to the plant host (Hestrin et al., 2019; Pandit, et al., 2022; Duan et al., 2025). In addition, carbon and mineral nutrient exchange between AMF and hyphospheric bacteria is believed to be critical for the efficient acquisition of mineral nutrients from soil by AMF (Duan et al., 2024). Therefore, in studies involving live soil, both cooperative and competitive interactions between AMF and the broader endo/rhizobiome are most likely at play. In the current study (Experiment 1), AMF colonization was generally lower in G.11 roots grown in live vs. pasteurized soil when comparing otherwise identical treatments. Antagonistic interactions with soil-borne microbes in live soil may have influenced the capacity of G.11 rootstocks to form associations with AMF. In support of these findings, an earlier study conducted with apple found that AMF colonization levels were lower in the roots of replant-affected trees than in those of healthy apple trees (Caruso et al., 1989).

An interesting area, which has not received much attention, is the role of specific rootstock genotype-AMF associations in mediating resistance or tolerance to apple replant disease. While tolerance refers to a plant’s ability to grow and produce despite being susceptible to pathogen infection and colonization, resistance indicates a plant’s ability to limit infection or subsequent proliferation of the pathogen (Somera and Mazzola, 2022). In this study, even though *R. irregularis* likely played a central role in enhancing water and nitrogen uptake as G.11 root systems became compromised due to replant pressure, pathogen levels in root tissue were not significantly different between *R. irregularis* inoculated vs. uninoculated treatments. This suggests, *R. irregularis* inoculation may have compensated for water/nutrient losses caused by replant pathogens via hyphal-mediated transfer and/or other mechanisms, allowing the plant to better tolerate infection (without direct pathogen suppression).

In Experiment 2, the potential of select AMF–rootstock associations to improve resistance to the apple replant pathogen *Rhizoctonia solani* AG-5 was investigated; micropropagated plantlets were colonized by AMF and subsequently exposed to the pathogen in pasteurized orchard soil. Results showed that AMF can directly influence susceptibility to *R. solani* AG-5 in apple. This protective effect, however, varied according to the particular AMF species x host genotype combination: *C. etunicatum* (but not *C. claroideum*) significantly reduced subsequent infection by *R. solani* in G.41 (but not G.890), relative to uninoculated controls. This outcome (G.41 x *C. etunicatum*) differs from those of Experiment 1 (G.41 x *R. irregularis*), in which data from pathogen-specific qPCR assays (including *R. solani*) pointed to AMF-mediated stress/disease *tolerance*.

*R. solani* has an exceptionally wide host range (> 200 plant species) and the ability of AMF to effectively mitigate soilborne disease caused by this pathogen (in sterilized substrate) has been documented in a variety of cropping systems including potato (Yao et al., 2003), cucumber (Aljawasim et al., 2020), and watermelon (Wu et al., 2021). Despite that, few studies have provided evidence of AMF directly limiting pathogen establishment in root tissue (i.e., resistance). This distinction is critical for interpreting AMF efficacy, disease dynamics, context-dependent trade-offs, and/or potential impacts of AMF interventions. As covered in more detail earlier, the extent to which AMF colonization can benefit host resistance in natural orchard systems containing a diversity of potentially pathogenic organisms is another area which is not well-addressed in current literature. Given that the protective response is likely to be governed by several factors including AMF species x host genotype compatibility, the question now becomes, “Within what environmental contexts/agronomic constraints are these benefits likely to be reliably realized?”.

There are always caveats and challenges with AMF studies. Working with commercially-available, nursery-derived rootstocks (with pre-established microbial communities) in live soil (as in Expt. 1) presents significant challenges. Experimental systems involving the use of “clean” micropropagated plantlets in sterilized substrate (as in Expt. 2) provide control but lack “real-world” ecological complexity that may influence plant-AMF interactions. Standard nursery practice involves growing rootstock material in stooling or layering beds containing a mixture of sawdust and orchard soil. In the fall, the rootstock material is removed from the beds, bundled, and put into in cold storage (1-2˚C) for the winter. During this dormancy period, root endophytes likely become inactive/dormant as well. This could explain why little to no AMF colonization was (microscopically) detected in uninoculated (no AMF) treatments 6-weeks post planting (Experiment 1; **Figure 2**). Although species-level identification of AMF by microscopic observation is not possible, root length colonization measurements provided strong evidence that the *R. irregularis* inoculum established relationships with new roots (faster than pre-existing AMF) and that plants were influenced by this association prior to the start of the water stress/nitrogen uptake portion of the experiment. In a separate study, which assessed several apple rootstock genotype/AMF interactions, *R. irregularis* and *C. etunicatum* were found to colonize apple roots more readily than other AMF tested, regardless of rootstock genotype (Cook et al., 2024). Nursery-derived AMF have been shown to serve as a significant source of inoculum in apple rootstocks (Zhang et al., 2025). By 10-weeks post planting, AMF colonization levels had increased in uninoculated (no AMF) x pasteurized treatments. Consequently, over the course of the experiment, all plants likely became actively colonized by pre-existing (nursery-derived) AMF to some degree.

While the research from this study highlights the potential benefits of tailoring specific AMF–rootstock genotype associations in tree fruit production systems, it also underscores both the complexity and context-dependency of the interactions and their functional benefits. Mycorrhizal-mediated tolerance/resistance to soilborne disease and water/nutrient stress in apple likely involves complex interactions between rootstock genotype, AMF species, microbial community context and environmental conditions. Untangling these complexities remains an important challenge in many high value cropping systems like apple, especially those that have high water requirements for production. The results of this study support the role of AMF as multifunctional symbionts capable of benefitting plant water and nutrient acquisition as well as defensive functions in apple production systems. In conclusion, predicting the multifaceted functions of specific apple rootstock/AMF combinations in the presence of other microorganisms is a complex but promising frontier and a necessary step toward harnessing the potential of AMF for more productive, resilient and sustainable cropping systems. Rather than utilizing a uniform systems approach, going forward, the results of this study stress the importance of developing locally-tailored strategies to AMF-based interventions, in which environmental conditions and orchard/nursery management strategies are considered together.

## Data Availability

The original contributions presented in the study are included in the article/**Supplementary Material**. Further inquiries can be directed to the corresponding author.

## Supplementary material

Supplementary Figure 1 Light microscopy image of *Rhizoctonia solani* isolated from *R. solani* AG-5 oat-infested culture plated onto 1/5^th^ PDA + ampicillin (100 µg/ml). The blue arrow highlights a region in which the septum is visible immediately after branch points. The red arrow shows a point of hyphal fusion. Both structures are indicative of *R. solani*.

Supplementary Figure 2 Volumetric soil water content in live (top) and pasteurized (bottom) soil treatments over the 1-month duration of the water stress experiment (6–10 weeks post-planting, 28 days; sensor data readings from solar noon). Blue and orange lines indicate well-watered (WW) and water-stressed (WS) treatments, respectively. Solid vs. dashed lines indicate non-inoculated vs. *R. irregularis*-inoculated treatments, respectively.

Supplementary Figure 3 Daily weight of the plant pots with trend lines (6–10 weeks post-planting). Each point represents the average of 7 replicate pots (per treatment). Water droplet icons indicate watering days. Well-watered (WW) treatments are shown in blue, while water-stressed (WS) treatments are colored orange. Solid circles/lines = no-AMF controls; open triangles/dashed lines = plants inoculated with *R. irregularis*.

Supplementary Figure 4 Bar graph showing total plant N content for all 8 treatments. The dotted line separates live vs. pasteurized soil treatments. Well-watered (WW) treatments are shown in blue, while water-stressed (WS) treatments are colored orange. Hatched bars represent treatments in which plants were colonized by *R. irregularis* prior to experiencing different watering regimes. Each point represents data from a single plant (n=6 for all treatment combinations, except Live x AMF x WS where n=5).

Supplementary Figure 5 Relative ^15^N enrichment of plant leaves, wood, and root tissue in G.11 rootstocks cultivated in live and pasteurized soil under different watering regimes. Well-watered (WW) treatments are shown in blue, while water-stressed (WS) treatments are colored orange. “AMF” indicates treatments in which plants were colonized by *R. irregularis* prior to experiencing the different watering regimes. Error bars represent the standard error (n=6 for all treatment combinations, except Live x AMF x WS where n=5).

Supplementary Figure 6 Box and whisker plot depicting relative abundance of root-associated *R. irregularis* DNA on G.11 apple rootstocks subjected to 4 weeks of 40% water stress (WS) compared to well-watered (WW) rootstocks cultivated in either live or pasteurized replant soil. ddCT values were determined by comparison of the difference between CT values for the apple reference target gene and the *R. irregularis* target between treatments. Mean and median values are represented by an x and line, respectively.

Supplementary Figure 7 Box and whisker plot depicting a 46-fold higher relative abundance of root-associated *R. intraradices* DNA on G.11 apple rootstocks subjected to 4 weeks of 40% water stress compared to well-watered rootstocks in live replant soil. ddCT values were determined by comparison of the difference between CT values for the apple reference target gene and the *R. intraradices* target between treatments.

Supplementary Figure 8 Abundance of *Cylindrocarpon* (*Ilyonectria*) spp. (left) and *Rhizoctonia solani* (right) detected by qPCR in G.11 apple root tissue. Data is shown according to treatment and soil type (open circles = pasteurized orchard soil; filled circles = live orchard soil). Treatments designated with asterisks differ significantly in pathogen DNA content between soil types (two-way ANOVA followed by Šidák’s multiple comparisons test; p < 0.01). Prior to analysis, qPCR were normalized to pg DNA per ng input DNA.
