# Supplementary material for: Assessing functional benefits of specific apple rootstock/AMF associations within context-dependent frameworks
Source: Front Plant Sci. 2026 Sep 24;17:1914090. doi: 10.3389/fpls.2026.1914090 (PMC13662920; doi:10.3389/fpls.2026.1914090)
Supplement: Supplementary file 1 [file SupplementaryFile1.pdf]

Supplementary Figures

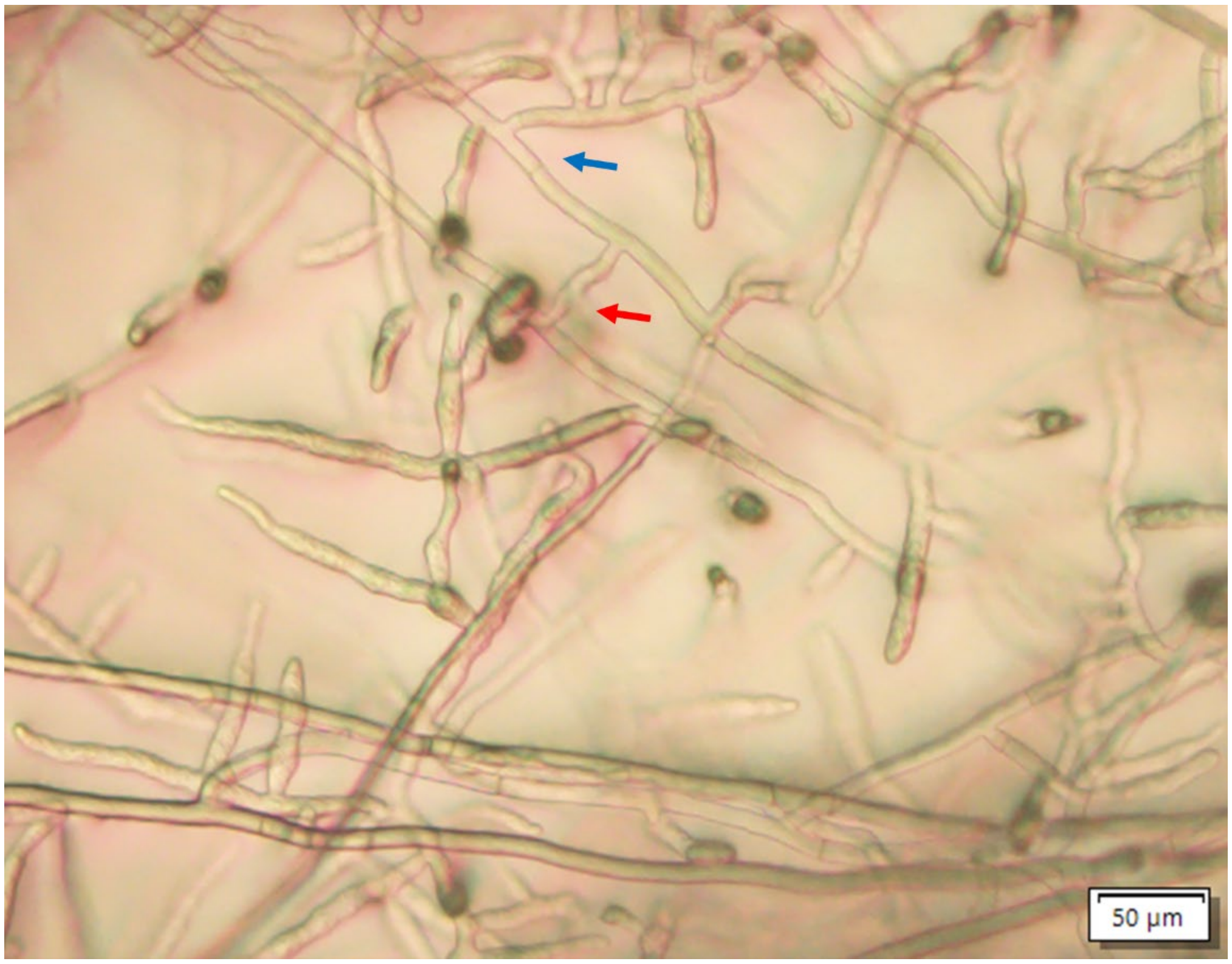

Supplementary Figure 1. Light microscopy image of *Rhizoctonia solani* isolated from *R. solani* AG-5 oat-infested culture plated onto 1/5th PDA + ampicillin (100 µg/ml). The blue arrow highlights a region in which the septum is visible immediately after branch points. The red arrow shows a point of hyphal fusion. Both structures are indicative of *R. solani*.

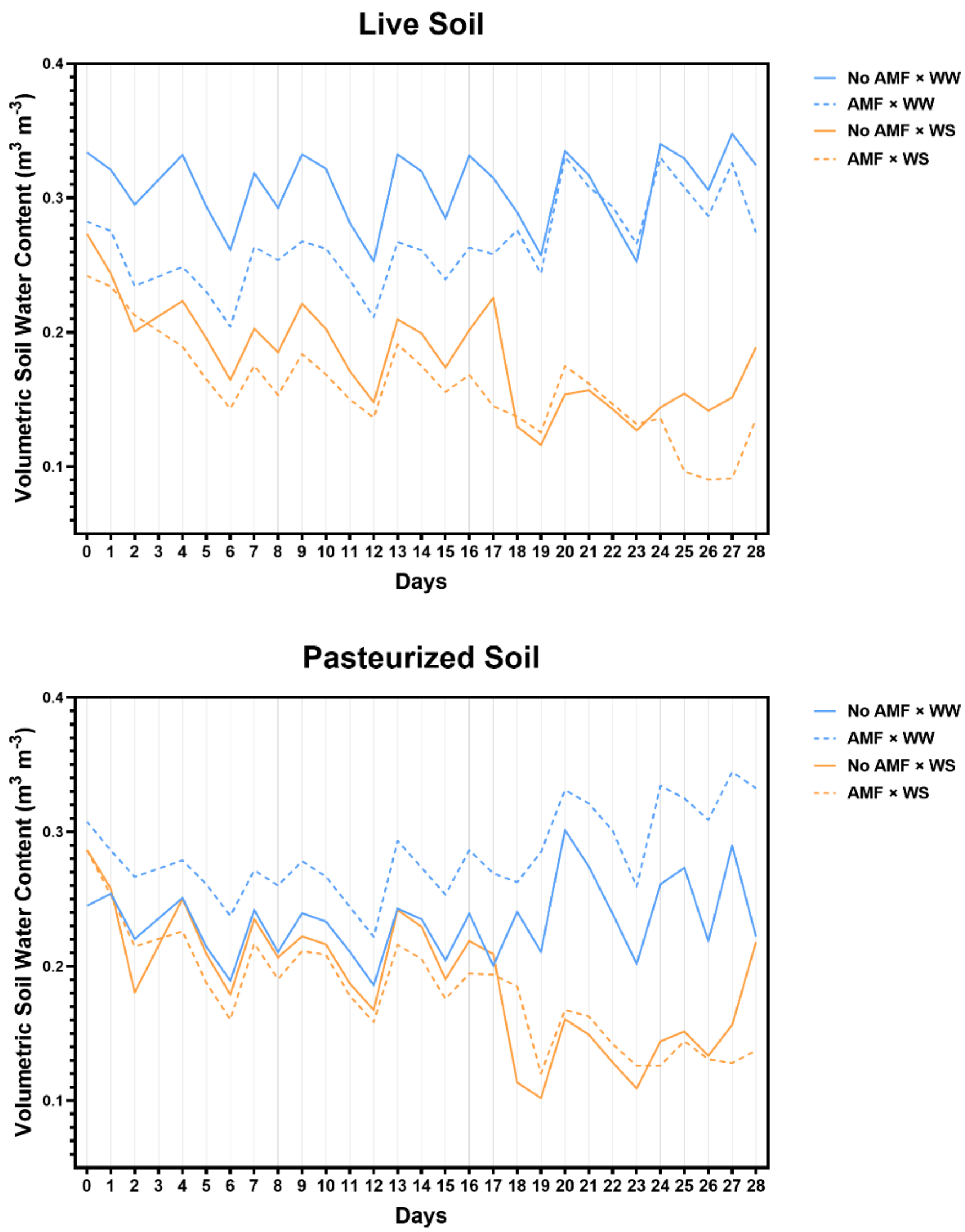

Supplementary Figure 2. Volumetric soil water content in live (top) and pasteurized (bottom) soil treatments over the 1-month duration of the water stress experiment (6-10 weeks post-planting, 28 days; sensor data readings from solar noon). Blue and orange lines indicate well-watered (WW) and water-stressed (WS) treatments, respectively. Solid vs. dashed lines indicate non-inoculated vs. *R. irregularis*-inoculated treatments, respectively.

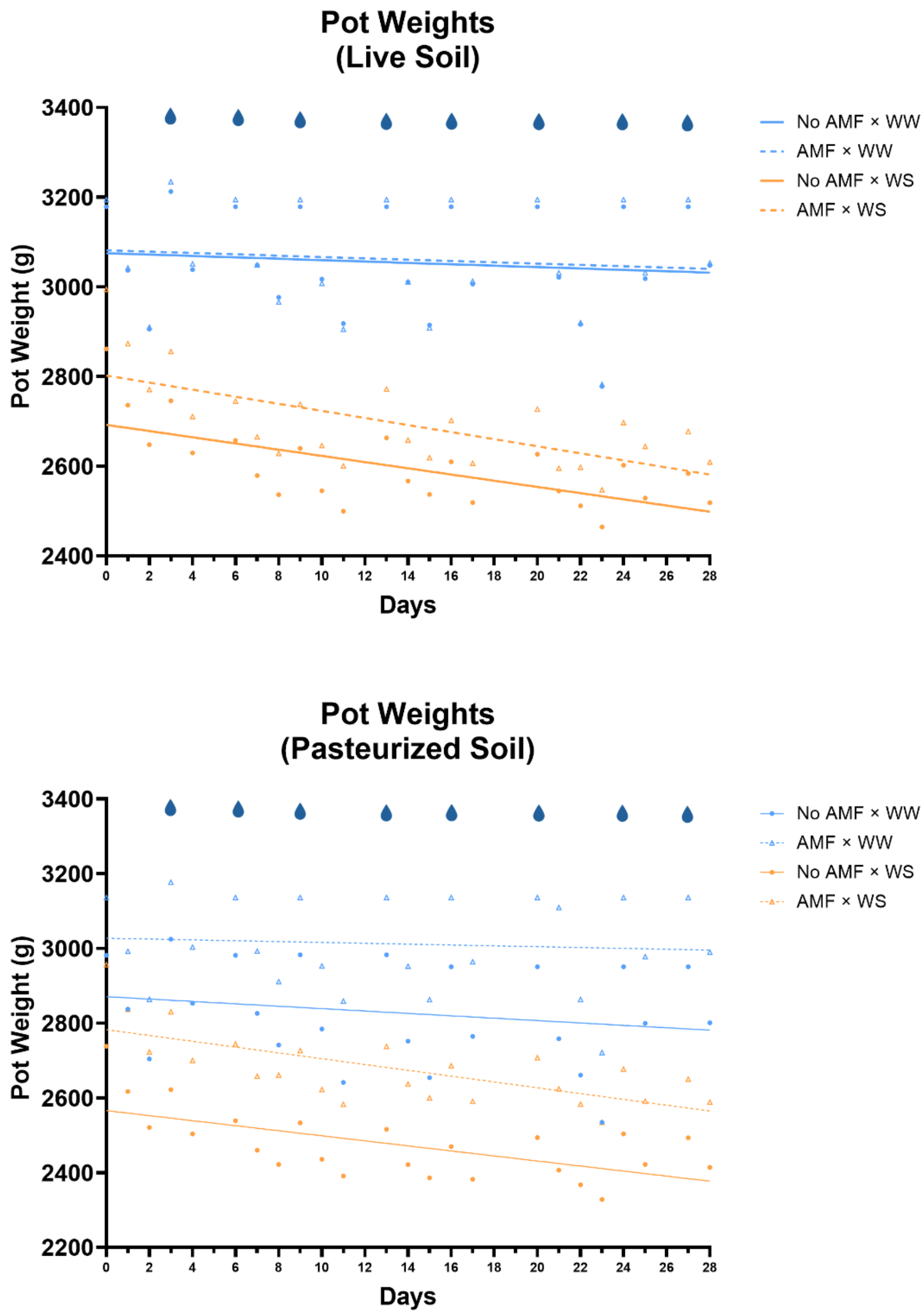

Supplementary Figure 3. Daily weight of the plant pots with trend lines (6-10 weeks post-planting). Each point represents the average of 7 replicate pots (per treatment). Water droplet icons indicate watering days. Well-watered (WW) treatments are shown in blue, while water-stressed (WS) treatments are colored orange. Solid circles/lines = no-AMF controls; open triangles/dashed lines = plants inoculated with *R. irregularis*.

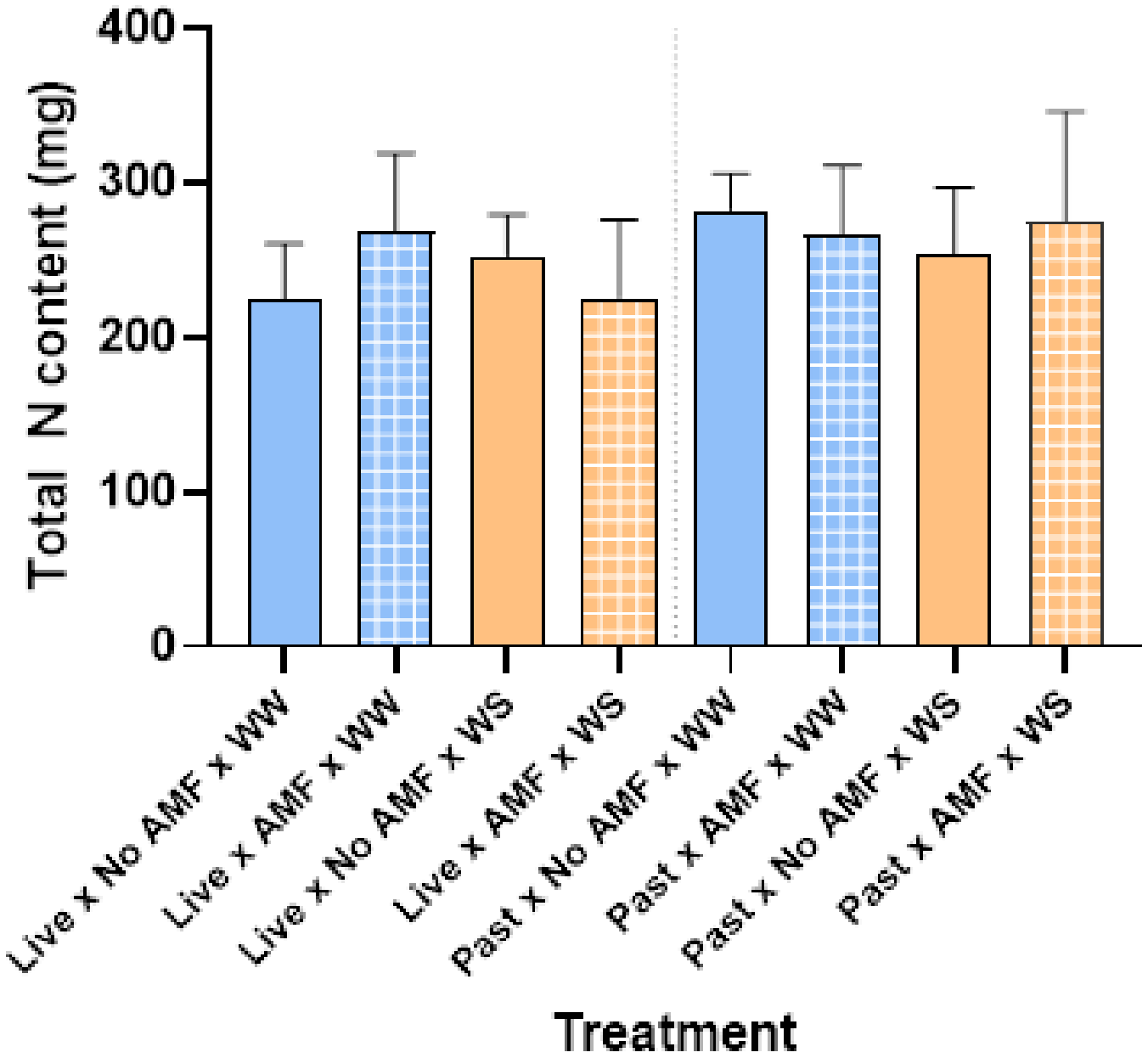

Supplementary Figure 4. Bar graph showing total plant N content for all 8 treatments**.** The dotted line separates live vs. pasteurized soil treatments. Well-watered (WW) treatments are shown in blue, while water-stressed (WS) treatments are colored orange. Hatched bars represent treatments in which plants were colonized by *R. irregularis* prior to experiencing different watering regimes. Each point represents data from a single plant (n=6 for all treatment combinations, except Live x AMF x WS where n=5).

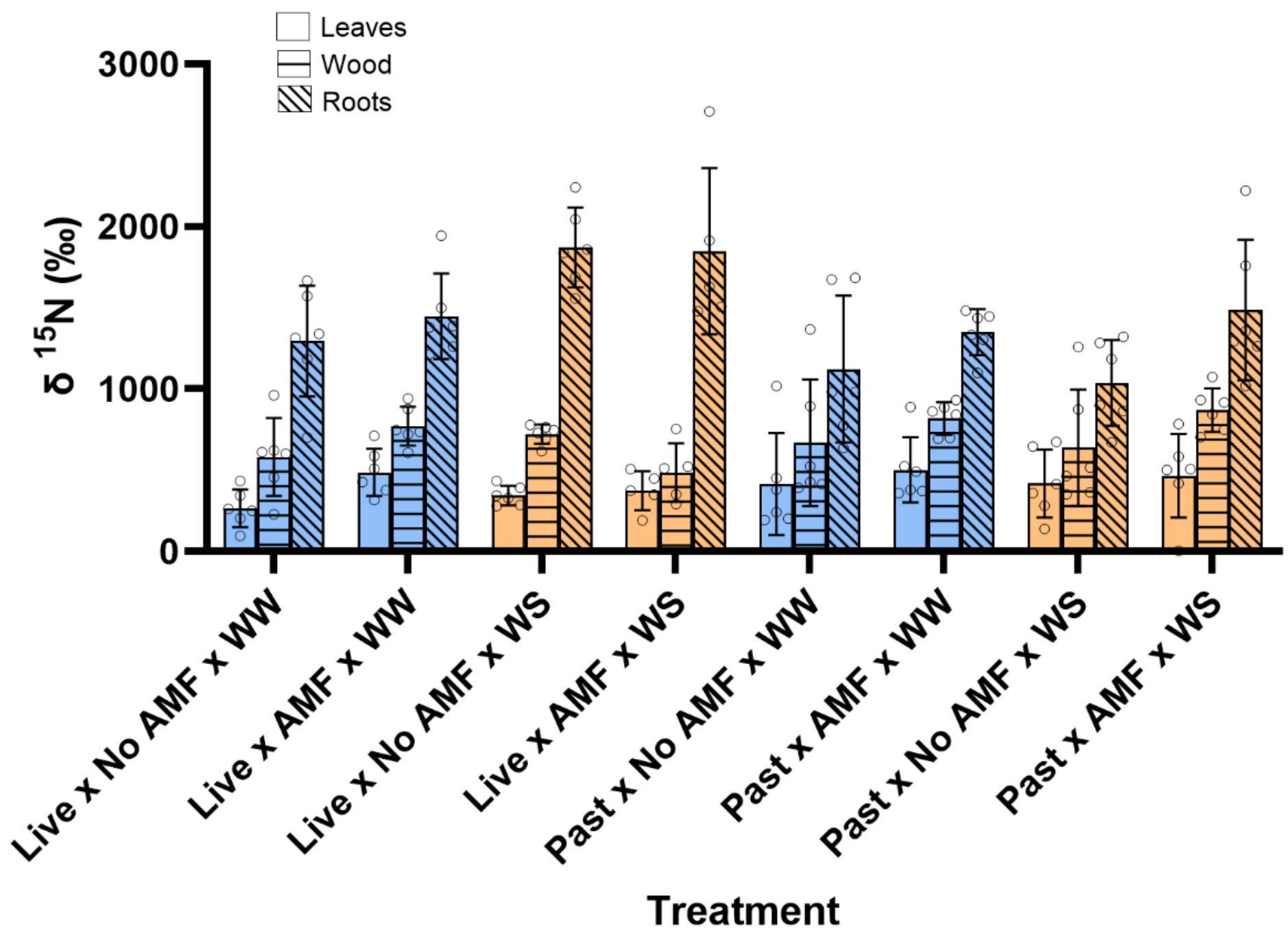

Supplementary Figure 5. Relative ^{15}N enrichment of plant leaves, wood, and root tissue in G.11 rootstocks cultivated in live and pasteurized soil under different watering regimes. Well-watered (WW) treatments are shown in blue, while water-stressed (WS) treatments are colored orange. "AMF" indicates treatments in which plants were colonized by *R. irregularis* prior to experiencing the different watering regimes. Error bars represent the standard error (n=6 for all treatment combinations, except Live x AMF x WS where n=5).

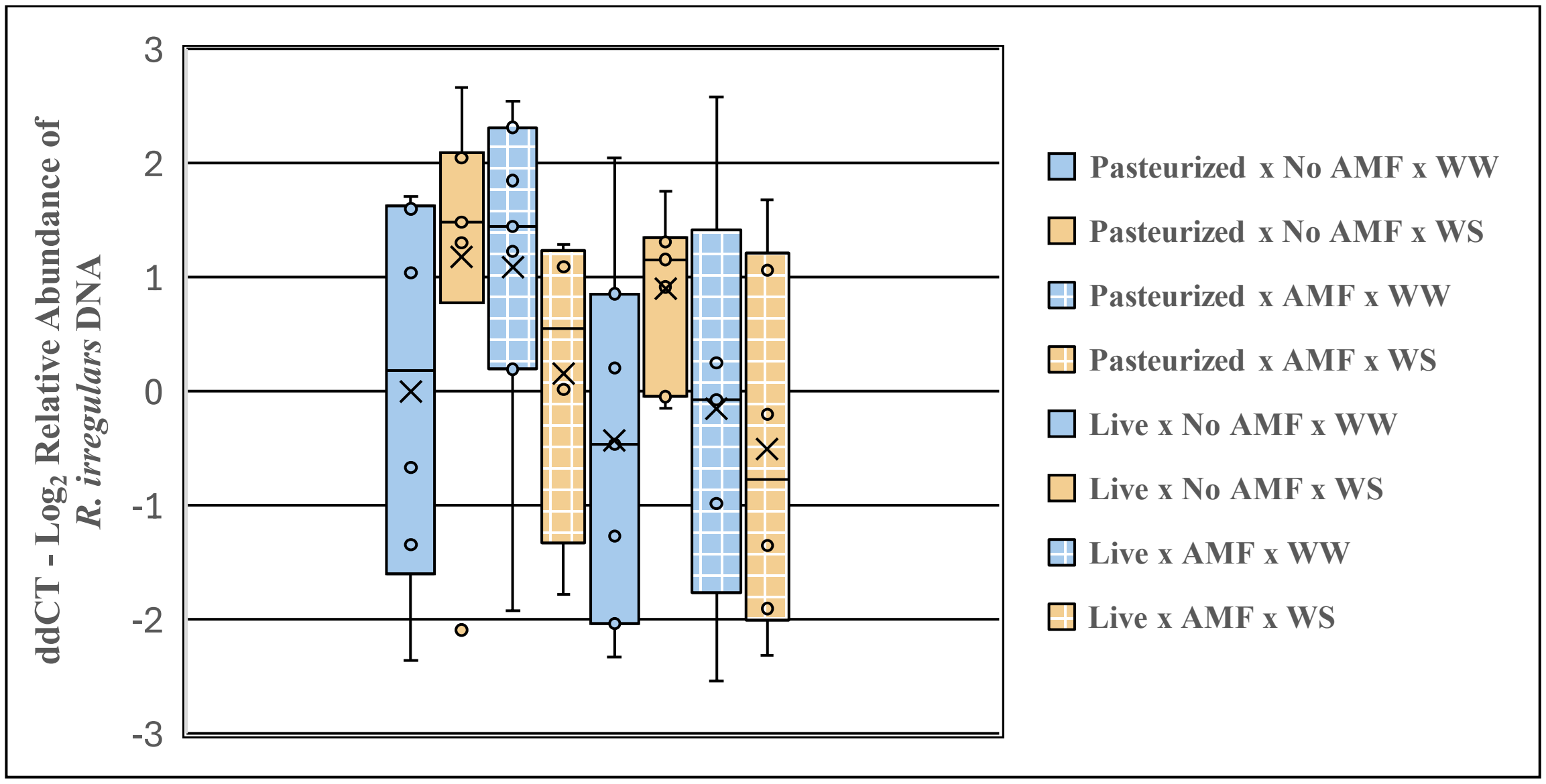

Supplementary Figure 6. Box and whisker plot depicting relative abundance of root-associated *R. irregularis* DNA on G.11 apple rootstocks subjected to 4 weeks of 40% water stress (WS) compared to well-watered (WW) rootstocks cultivated in either live or pasteurized replant soil. ddCT values were determined by comparison of the difference between CT values for the apple reference target gene and the *R. irregularis* target between treatments. Mean and median values are represented by an x and line, respectively.

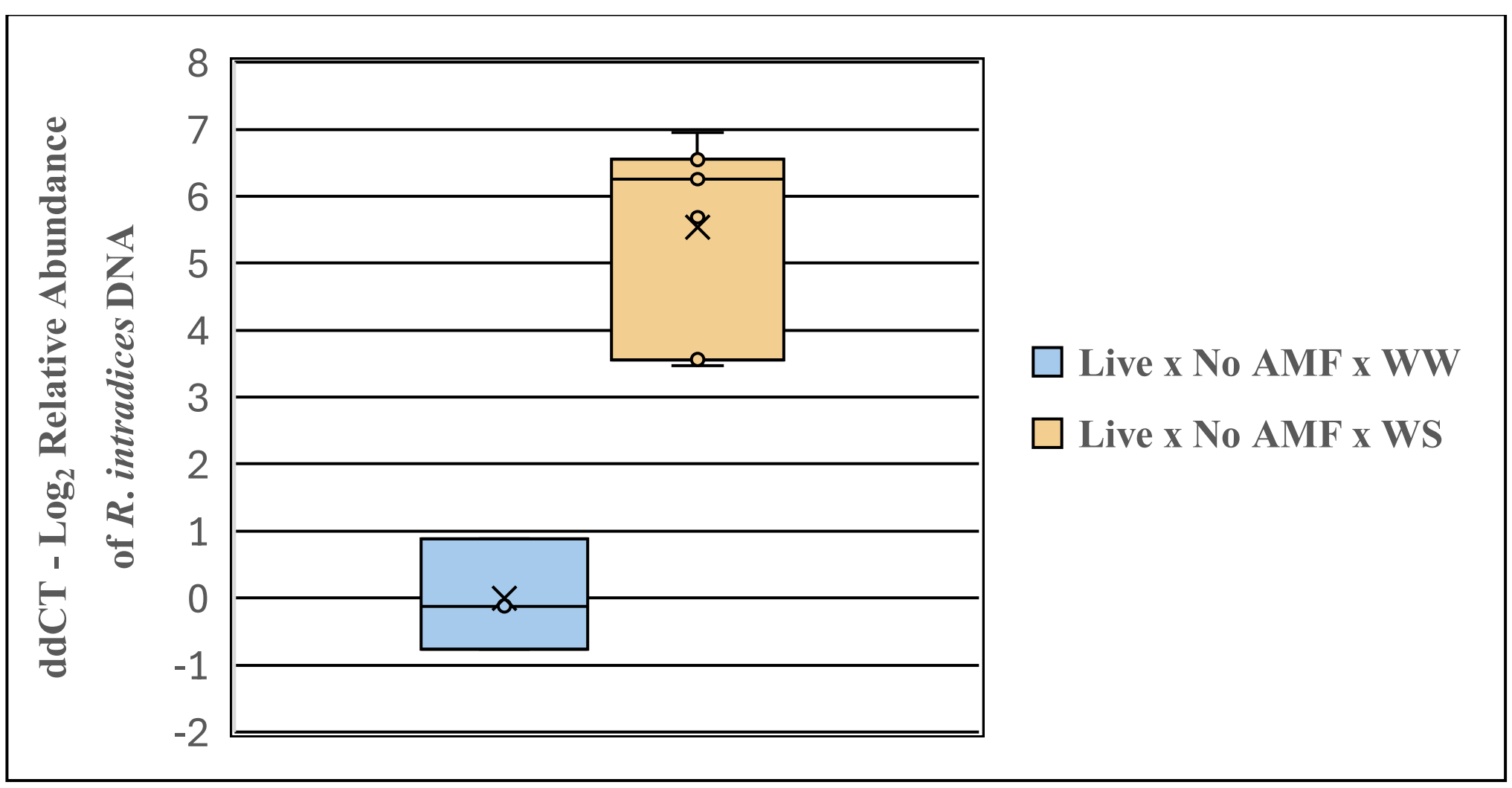

Supplementary Figure 7. Box and whisker plot depicting a 46-fold higher relative abundance of root-associated *R. intraradices* DNA on G.11 apple rootstocks subjected to 4 weeks of 40% water stress compared to well-watered rootstocks in live replant soil. ddCT values were determined by comparison of the difference between CT values for the apple reference target gene and the *R. intraradices* target between treatments.

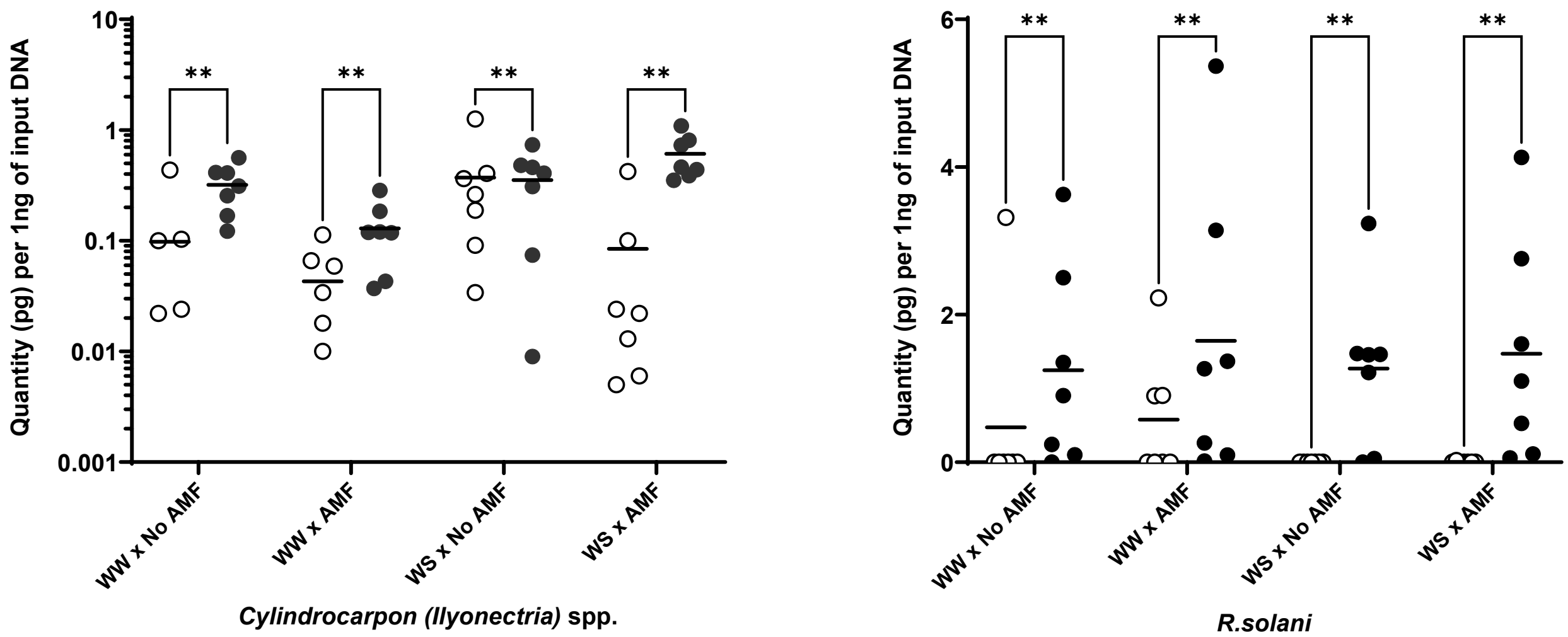

Supplementary Figure 8. Abundance of *Cylindrocarpon* (*Ilyonectria*) spp. (left) and *Rhizoctonia solani* (right) detected by qPCR in G.11 apple root tissue. Data is shown according to treatment and soil type (open circles = pasteurized orchard soil; filled circles = live orchard soil). Treatments designated with asterisks differ significantly in pathogen DNA content between soil types (two-way ANOVA followed by Šidák's multiple comparisons test; $p < 0.01$). Prior to analysis, qPCR were normalized to pg DNA per ng input DNA.
